# Screening for biomarkers reflecting the progression of *Babesia microti* infection

**DOI:** 10.1186/s13071-018-2951-0

**Published:** 2018-07-03

**Authors:** Bin Xu, Xiu-Feng Liu, Yu-Chun Cai, Ji-Lei Huang, Rui-Xiang Zhang, Jun-Hu Chen, Xun-Jia Cheng, Xia Zhou, Xue-Nian Xu, Yan Zhou, Ting Zhang, Shen-Bo Chen, Jian Li, Qun-Feng Wu, Cheng-Song Sun, Yong-Feng Fu, Jia-Xu Chen, Xiao-Nong Zhou, Wei Hu

**Affiliations:** 1 National Institute of Parasitic Diseases, Chinese Center for Disease Control and Prevention, WHO Collaborating Center for Tropical Diseases; National Center for International Research on Tropical Diseases, Ministry of Science and Technology; Key Laboratory of Parasite and Vector Biology, National Health and Family Planning Commission, Shanghai, People’s Republic of China; 2 Department of Microbiology and Microbial Engineering, School of Life Sciences, Fudan University, Shanghai, People’s Republic of China; 3 Institute of Biomedical Sciences, Department of Medical Microbiology and Parasitology, School of Basic Medical Sciences, Fudan University, Shanghai, People’s Republic of China; 4 Department of Parasitology, Medical College of Soochow University, Suzhou, People’s Republic of China

**Keywords:** *Babesia microti*, Strain ATCC®PRA-99TM, Mice model, Cell free expression system, Protein microarray, Screening biomarker, Expression and evaluation of proteins

## Abstract

**Background:**

Babesiosis is caused by the invasion of erythrocytes by parasites of the *Babesia* spp*. Babesia microti* is one of the primary causative agents of human babesiosis. To better understand the status of the disease, discovering key biomarkers of the different infection stages is crucial.

**Results:**

This study investigated *B. microti* infection in the mouse model from 0 to 270 days post-infection (dpi), using blood smears, PCR assays and ELISA. PCR assays showed a higher sensitivity when compared to microscopic examination. Specific IgG antibodies could be detected from 7 days to 270 dpi. Two-dimensional electrophoresis was combined with western blotting and mass spectrometric analysis to screen for specific reactive antigens during both the peak parasitaemia period (7 dpi) and IgG antibody response peak period (30 dpi) by the infected mice plasma. The 87 positive reactive proteins were identified and then expressed with the wheat germ cell-free system. Protein microarrays of all 87 targeted proteins were produced and hybridized with the serial plasma of infected mice model. Based on the antigen reaction profile during the infection procedure, 6 antigens were selected and expressed in *Escherichia coli*. Due to an early response to IgM, lower immunoreactivity levels of IgG after two months and higher immunoreactivity level IgG during nine months, four recombinant proteins were selected for further characterization, namely rBm2D97(CCF75281.1), rBm2D33(CCF74637.1), rBm2D41(CCF75408.1) and rBm7(CCF73510.1). The diagnostic efficacy of the four recombinant protein candidates was evaluated in a clinical setting using babesiosis patient plasma. The rBm2D33 showed the highest sensitivity with a positive rate of 62.5%. Additional characterization of the two candidate proteins using a mouse vaccination assay, demonstrated that rBm2D41 could reduce peak parasitaemia by 37.4%, indicating its efficacy in preventing severe babesiosis.

**Conclusions:**

The detection technologies of microscopic examination, PCR assays and antibody tests showed different sensitivities and accuracy during the different stages of *B. microti* infection. Antibody detection has a unique significance for *B. microti* infection in the asymptomatic stages. Using immunoreactivity profiles, biomarkers for disease progression were identified and represent useful information for future the diagnosis and vaccine development for this serious disease of public health significance.

**Electronic supplementary material:**

The online version of this article (10.1186/s13071-018-2951-0) contains supplementary material, which is available to authorized users.

## Background

The zoonotic parasitic disease, babesiosis, is caused by the intraerythrocytic protozoans *Babesia* spp. *Babesia* spp. are usually transmitted by *Ixodes* ticks, blood transfusions or from mother to child transplacentally [1–3]. Among more than 100 identified *Babesia* species, only a few have been reported to infect humans, including *Babesia microti* and *B. microti*-like organisms, *B. duncani* and *B. duncani-*type organisms, *B. divergens* and *B. divergens*-like organisms, and *B. venatorum* [4–7]. Babesiosis became a nationally recognized disease in January 2011 in the USA. *Babesia microti* has been implicated in the majority of clinical cases in the USA; including both tick-borne and transfusion-transmitted babesiosis (TTB) [8]. This is reflected in a survey of TTB over the period of 1979-2009, where *B. microti* was attributed as the infectious species [8]. The dynamics of parasitaemia and immune responses in the rhesus macaque model, demonstrated that even very low infectious doses can result in TTB, and the low-grade or asymptomatic parasitemia could persist for long periods [9]. Since there is a lack of a Food and Drug Administration (FDA) approved blood screening, *B. microti* has become a high-risk pathogen that is transmitted by blood transfusions in the USA [6].

Different severities and symptoms are observed in *B. microti*-infected patients. It was always asymptomatic in healthy people, but among immune-deficient patients such as people who have undergone a splenectomy, HIV infected, or elderly, the infection can relapse and potentially become lethal [6, 10–13]. *Babesia microti* infections in immuno-intact primates are usually self-limiting, and most clinical cases recover without intervention [14]. However, low levels of parasitaemia have been observed in rat and monkey infection models [15, 16]. In the *B. microti-*infected CBA mice model, parasitaemia levels gradually increased and reached peak levels in the acute phase, followed by levels quickly declining and persisting with very low parasitaemia. While parasitaemia was undetectable or cleared, antibody levels peaked and entered a chronic phase [17].

In the investigation of *B. microti* infection in blood donors of nonendemic and endemic areas in the USA, higher numbers of positive samples were detected using antibodies when compared to the use of PCR methods [18]. However, the risk of TTB for antibody-positive donors was confirmed and highlighted, no matter if they were PCR-positive or not [19]. From these observations, it is clear that antibody testing is crucial for the screening of the cases infected by *B. microti*. Further study of *B. microti-*related protective immune mechanisms, indicated that IgM antibodies first showed a response against the parasite in the acute phase and that specific IgG antibodies were related to decrease parasitaemia in the blood [17, 20–22]. However, the immunoreactivity profiles of *B. microti* infection during the acute to chronic phase remain unclear. Thus, it would be important to find the biomarkers for detection of the disease progression and identify the key antigens related to immune protection or the self-limiting phenomenon in immune-intact individuals.

In the current study, the *B. microti*-infected mice model was established, and specific reactive antigens identified during the peak parasitaemia period, also antibody levels were analyzed and expressed. A high-throughput proteomic analysis was then carried out to obtain immunoreactivity profiles. Finally, candidate molecules were screened for use as clinical diagnostics, and as potential vaccines evaluated in an animal model.

## Methods

### Sample collection

Eight plasma samples were collected from patients with babesiosis from Fujian, Guangdong, Xinjiang provinces and Shanghai in the P.R. China. All patients were experiencing fever, and six patients had hepatosplenomegaly. All blood samples were PCR-positive, five of which were microscopically positive (Additional file 1: Table S1). Two hundred samples collected from febrile patients in Tengchong, Yunan Province, a malaria endemic area, were microscopically negative. We also collected ten patient plasma of *P. falciparum* and *P. vivax* that were diagnosed by parasitological detection (blood smears) as malaria infection from Yunnan Province. Plasma samples from unexposed individuals, used as controls, were collected in medical college at Soochow University, Zhejiang Province.

### Establishing the BALB/c mice model with *B. microti*

*Babesia microti* strain ATCC®PRA-99TM, was obtained from the Institute of Laboratory Animal Sciences, Chinese Academy of Medical Sciences (CAMS). Ten female BALB/c mice aged six weeks were inoculated with 100 μl of donor blood with 1.8 × 10^7^ *B. microti*-infected erythrocytes by intraperitoneal injection. Anticoagulant blood (200 μl) was collected before infection as a control. At each time point in the study, the same dose of anticoagulant blood was collected from the caudal vein of infected mice at 3, 7, 14, 21, 30, 60, 120, 150 and 270 days post-infection (dpi). Erythrocytes and plasma were separated from all blood samples by standard methods, for molecular detection and antigen screening and evaluation.

#### Staining and microscopy examination

Blood samples were extracted from mice tail tips to prepare thin smears on microscope slides every dpi until day 30 and for each time point in the study. All smears were air-dried, fixed in absolute methanol, stained with Giemsa stain (Baso, Zhuhai, China), and then counted under ×1000 magnification by a bright-field microscope (Nikon, Tokyo, Japan). Five thousand erythrocytes were scanned for each sample, and the number recorded of the erythrocytes infected with *B. microti*.

#### Nucleic acid testing

Genomic DNA was extracted from erythrocytes of anticoagulant blood using the DNeasy Blood & Tissue Kit (Qiagen, Shanghai, China). A specific fragment of *B. microti 18S* rRNA [23] was set as the detecting target fragment, and the first-round primers were CRYPTORN (5'-GAA TGA TCC TTC CGC AGG TTC ACC TAC-3') and CRYPTOFL (5'-AAC CTG GTT GAT CCT GCC AGT AGT CAT-3'). The second-round primers were Babl (5'-GTC TTA GTA TAA GCT TTT ATA CAG CG-3') and Bab4 (5'-GAT AGG TCA GAA ACT TGA ATG ATA CAT CG-3'). Nested PCR was performed in a 25 μl reaction containing 5 μl buffer, 2 μl dNTP mixture, 1 μl of each primer, 0.5 μl DNA polymerase (Takara R050A PrimeSTAR, Kanagawa, Japan) and 2 μl DNA template. The reactions were performed under the following conditions: the first-round amplification: 98 °C for 3 min, followed by 35 cycles of 10 s at 98 °C, 15 s at 60 °C, 2 min at 68 °C, and a final extension of 68 °C for 10 min. The second-round amplification was carried out in the same manner as the first except that the DNA template was a 1:100 dilution of the first-round PCR product and the degenerate temperature was 55 °C. The PCR reactions were performed using PTC-200 PCR System (BIO-RAD). The PCR product specificity was verified by electrophoresis, sequencing and NCBI/BLASTn.

#### Antibody level detection

Anticoagulation blood was collected from ten infected BALB/c mice when parasitaemia reached approximately 60%. The parasites were isolated by a previously reported method [24]. Crude *B. microti* proteins were produced from the supernatant of purified parasites homogenized in PBS by ultrasound. Protein concentrations were determined using the Bradford method. The proteins were stored at -20 °C until needed. Crude protein extractions were diluted in carbonic acid buffer to a final concentration of 1 μg/ml. All of plasma samples from 10 inoculated individuals were pooled together, diluted 50-fold in PBST and incubated with HRP-conjugated goat anti-mouse IgG (Sigma-Aldrich). Absorbance of each well at 450 nm was measured by MTP-500 micro plate reader (Corona Electric, Ibaraki-Ken, Japan).The cut-off value was defined as the mean value plus 2.1 standard deviations (SD) of the mean optical density (OD).

### Preliminary screening immunogenic antigens

Crude *B. microti* protein extractions (100 μg and 800 μg) were loaded onto analytical and preparative gels and were separated by two-dimensional electrophoresis (2-DE). Six gels were carried out simultaneously under the same conditions to keep the experiment uniform in distribution and size of protein spots. Two of the gels were treated with silver stain and the others divided into two groups to perform immunoblots.

Proteins of the same four gels were electrotransferred simultaneously to homologous hybond-C nitrocellulose membranes (GE Amersham Biosciences, Wauwatosa, USA) for 90 min at 220 mA, then incubated with 3% BSA in PBS overnight at room temperature. After blocking, membranes were washed three times in PBST, followed by incubation in 7 dpi or 30 dpi plasma samples (both 1:100 in PBST) and their negative control (collected before inoculation) at room temperature for 2 h. HRP conjugated goat anti-mouse IgG (Sigma-Aldrich, St. Louis, USA, diluted 1:1000 in PBST) was added to membranes, and were developed with H_2_O_2_ and 3, 3'-Diaminobenzidine(DAB). Images of immunblots were acquired by Bio-Rad GS710 scanner and analyzed using Image Master Software (Amersham). Specific spots were observed on the membrane and were matched with the silver stained gels followed by identification by liquid chromatography-mass spectrometry (LC/MS-MS) as previously described [25]. The acquired mass spectra files were converted to mzXML files using software [26], and deposited in the ProteomeXchangeConsortium [27] *via* the PRoteomicsIDEntifications (PRIDE)v2.4.2 [28] partner repository with the dataset identifier IPX0001196000.

### Acquisition of immunoreactivity profiles

#### Cloning and expression

To efficiently clone and express the candidates (Additional file 2: Table S2), the genes were divided into gene fragments by SMART (http://smart.embl-heidelberg.de/). All genes were PCR amplified as previously reported [29]. Gene-specific primers were designed by Primer premier 5.0 and In-Fusion PCR primers using In-Fusion Primer Design Tool (Additional file 3: Table S3). The presence of a signal peptide was determined by SignalP 4.1[30] and GPI anchor determined by both GPI Modification Site Prediction [31] and PredGPI [32], were excluded from the gene expression constructs. Genomic DNA of *B. microti* was used to amplify target genes with the same amplification system as described above. The pEU-His vector (derived from pEU, CellFree Sciences, Matsuyama, Japan) linearized by double digestion with restriction enzymes *Xho*I and *BamH*I (Takara, Kanagawa, Japan) was used in a high-throughput manner as described in a previous report [33].

All successfully cloned fragments were expressed by the bilayer translation reaction method with the wheat germ cell-free (WGCF) system [34]. *Babesia microti* proteins were separated by sodium dodecyl sulfate polyacrylamide gel electrophoresis (SDS-PAGE) under denaturing conditions. Separated proteins were transferred to Polyvinylidene Fluoride (PVDF) membranes (Millipore, Billerica, MA, USA), followed by blocking with 3% BSA in PBS. Penta-His antibody (Qiagen, Duesseldorf, Germany) diluted 1:2000 in PBS and the same dilution of secondary HRP-conjugated goat anti-mouse IgG (Pierce, Waltham, MA, USA) were used to detect His-tagged recombinant proteins. The immunoblots were stained using H_2_O_2_ and DAB. The results were recorded by a ScanJet 5300C Scanner (Hewlett-Packard, Palo Alto, USA).

#### Preparing protein microarray and acquiring immunoreactivity profiles

OPEpoxy glass slides (75 × 25 mm) were used for the carrier of protein arrays (CapitalBio, Beijing, China) and Teflon fence with 12 holes (6 × 2, diameter of 9 mm), in which a protein microarray would be formed, were attached to the glass slides to prepare amine arrays. *Babesia microti* proteins identified by 2-DE and Bm7(CCF73510.1) from immunoscreening of cDNA library of *B. microti* [35] were twice spotted into a fence of the arrays and incubated for 30 min at room temperature. BMSA [36, 37] was amplified from *B. microti* PRA99 strain cDNA and its recombinant protein rBmSA1 was expressed in *E. coli* BL21(DE3) strain as the positive control. The wheat germ lysate with empty vector served as the negative control. The array was firstly blocked with 3% BSA in PBST for 2 h at 37 °C and incubated at different time points with the pooled plasma samples from mice (1:50 in PBST) for 1 h at 37 °C. The reactions were visualized with Alexa Fluor 546 goat anti-mouse IgM and IgG (10 ng/μl, Invitrogen, Waltham, MA, USA) in PBST for 1 h at 37 °C and quantified by using ScanArray Express software version 4.0 (PerkinElmer, Waltham, MA, USA) as described previously [33]. The higher response of a probed protein against the normal plasma sample was considered to be positive when the relative ratio of signal intensity (SI) was > 2.0 as compared to the response to the infected mice plasma.

### Validation of effective antigens

#### Expression and purification of recombinant proteins

According to the results of the protein microarray, the DNA fragments encoding 2D33, 2D41, and 2D97, Bm7 were cloned into pET42a, pSmartI (Smart Life Sciences, Changzhou, China) and pET28a vectors (primer sequences are shown in Additional file 4: Table S4) and then expressed in an *E. coli* BL21(DE3) strain. The recombinant proteins, 2D33 and 2D41 were both soluble when expressed and designated rBm2D33, rBm2D41. While 2D97 and Bm7 were expressed as inclusion bodies and designated as rBm2D97 and rBm7. rBm2D33 was purified using glutathione sepharose 4B beads and rBm2D41 using His-Trap FF (GE Healthcare Life Sciences, Wauwatosa, USA). rBm2D97 and rBm7 were purified using the Micro Protein PAGE Recovery Kit (Sangon Biotech, Shanghai, China). The quantity of recombinant proteins was measured using a BCA protein assay kit (Thermo Fisher Scientific Inc., Waltham, USA). SUMO-tagged Bm2D41 was cleaved by SUMO protease (Solarbio, Beijing, China) according to the manufacturer’s instructions.

#### Evaluation of recombinant proteins as diagnostic antigens

The antigen-antibody reactions were evaluated using HRP-conjugated goat anti-mouse IgG (total IgG, Sigma-Aldrich) and IgM (μ-chain, Sigma-Aldrich). Responses to different time points of mice plasma samples were done by enzyme-linked immunosorbent assay (ELISA) as previously described. HRP-conjugated goat anti-human IgG (total IgG, Sigma-Aldrich) and IgM (μ-chain, Sigma-Aldrich) responses in eight babesiosis patients’ plasma for sensitivity evaluation of the diagnostic protein markers was performed to evaluate the cross-reactivity of proteins to *Plasmodium* infection. The plasma of ten *P. falciparum* and *P. vivax* patients were used for the determination of detection specificity.

The two hundred blood samples of febrile cases from a malaria endemic area were used in this study. The DNA of blood samples were extracted and detected by nested-PCR using specific primers of *B. microti*, *P. falciparum* and *P. vivax* [23, 38, 39]. The *Plasmodium* genus-specific primer pair was rPLU1 and rPLU5, a second PCR amplification was carried out with species-specific primer pairs rVIV1 and rVIV2 for *P. vivax* and rFAL1 and rFAL2 for *P. falciparum* (Additional file 5: Table S5). All plasma samples described above were tested against *B. microti* antigens: rBm2D33, rBm2D41, rBm7, rBmSA1 and crude proteins. rBm2D97 was uniquely detected with IgM (μ-chain) by ELISA, in which 45 plasma samples from unexposed subjects were used as negative controls. The positive cut-off value was calculated as the mean OD value of the normal controls plus 2 SD.

#### Vaccination and challenge infection

A total of 16 six-week-old female BALB/c mice were divided into four groups (*n* = 4). Purified rBm2D41 and rBm7, 50 μg emulsified in 100 ml of Freund’s complete adjuvant (Sigma-Aldrich), was inoculated into the back of the mouse by subcutaneous injection followed by two additional booster immunizations of 50 μg recombinant antigens with Freund’s incomplete adjuvant and PBS at 7-day intervals. The two mice control groups were set up with an adjuvant control group were PBS-immunized with Freund’s adjuvant and the no immunization group control group. One week after the final booster immunization, all mice were challenged with 1 × 10^7^ *B. microti*-infected erythrocytes [40, 41]. Parasitaemia was monitored every other day for 30 days by examining Giemsa-stained smears. The antibody level was monitored at 3, 7, 14, 21 and 30 dpi by ELISA.

### Statistical analysis

Parasitaemia was evaluated with the percent of infected erythrocytes. One-way analysis of variance (ANOVA), followed by Tukey’s multiple comparison test, was used to compare the parasitaemia mean values of all variables using GraphPad Prism software, version 5.0 (GraphPad, San Diego, USA)[42]. The heatmap was drawn using the Multi-array experiment viewer (MeV) software [43]. Hierarchical clustering analysis (HCA) was performed with R [44] using Ward linkage based on a distance matrix of the Pearson correlation of the samples and clustering was conducted through the *hclust* function in R using Euclidean distance[45].

## Results

### Dynamic changes of parasitaemia, PCR analysis and specific antibody level in BALB/c mice infected by *B. microti*

**Fig. 1:**
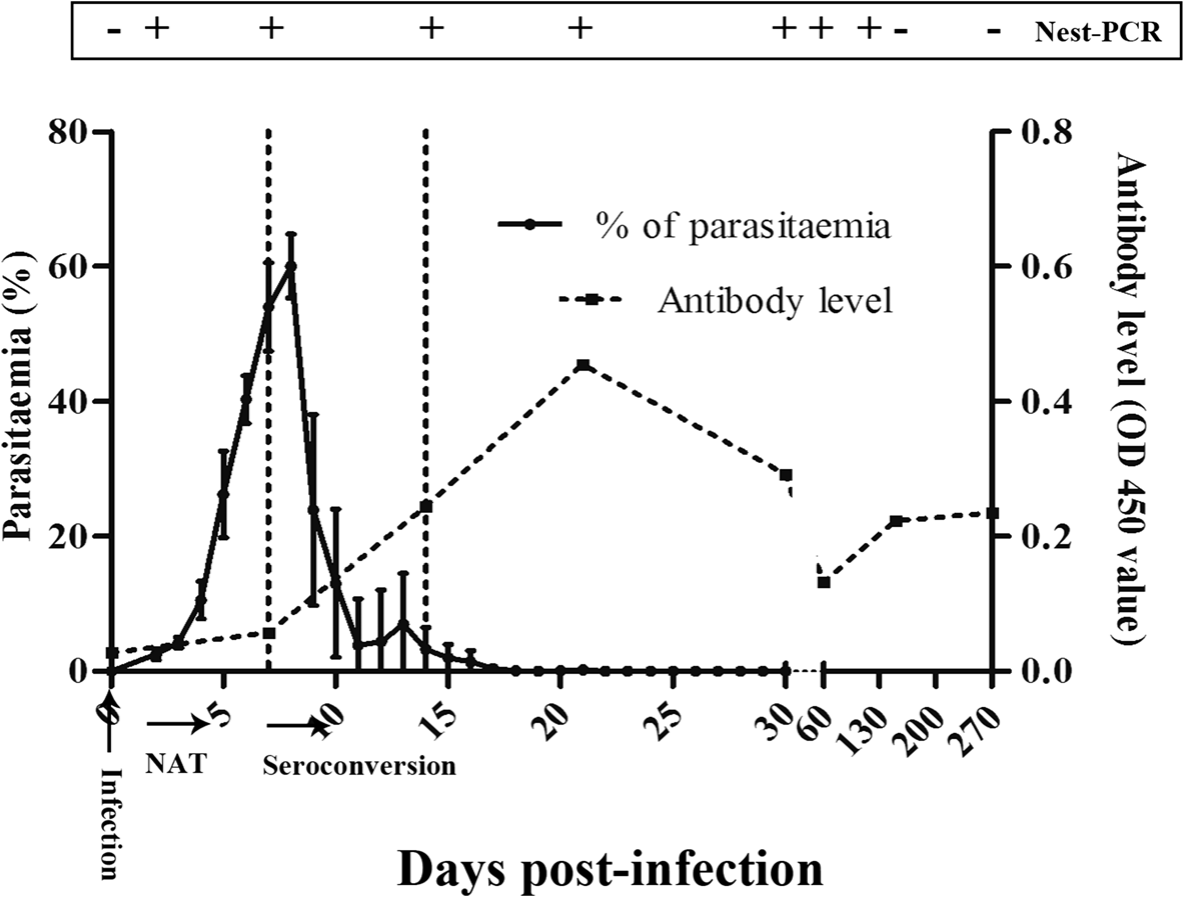
Dynamics of parasitaemia, PCR analysis and antibody level in BALB/c mice with *B. microti* model. Each point represents the mean ± SD

All 10 inoculated mice were infected successfully with *B. microti*. The dynamics of parasitaemia were observed by examination of thin blood smears (Fig. 1). The acute peak was detected with the average parasitaemia of 60% on Day 8 after experimental infection. After 20 dpi, few parasites were detected into the chronic stage of infection, and after 60 dpi, no parasitaemia was observed up to when the experiment was ended at 270 dpi (Table 1). Hemolysis, deeper urine color, and decreased vitality were observed in *B. microti-*infected BALB/c mice along with the increase of parasitaemia. *Babesia microti* DNA was successfully amplified from erythrocytes of the ten inoculated mice from 3 dpi to 60 dpi, only one positive was observed on 120 dpi (Table 1).

**Table 1:** Compared sensitivity of Giemsa-stained thin blood smears and nested PCR for *B. microti* infection in BALB/C mice during 9 months (+, positive; -, negative)

dpi	Microscopy (No. positive/No. tested)	Nested PCR (No. positive/No. tested)
0	- (0/10)	- (0/10)
3	+ (10/10)	+ (10/10)
7	+ (10/10)	+ (10/10)
14	+ (10/10)	+ (10/10)
21	+ (7/10)	+ (10/10)
30	+ (2/10)	+ (10/10)
60	+ (1/10)	+ (9/9)^a^
120	- (0/10)	+ (1/10)
150^b^	- (0/9)	- (0/9)
270^c^	- (0/6)	- (0/6)

*Abbreviation*: *dpi* days post-infection

^a^One sample was unavailable for PCR detection

^b^Nine mice alive on 150 dpi

^c^Six mice alive on 270 dpi

Antibody expression was determined by reaction intensity of crude *B. microti* proteins with mouse plasma from different infected periods as determined by ELISA (Fig. 1). The detectable antibody response appeared on Day 7 in this study, which indicated that there should be a window for antibody detection. The peak antibody titers were observed on Day 21, which was 2 weeks after the seroconversion and peak parasitaemia (7 dpi). The antibody levels gradually declined from 21 to 60 dpi and maintained a long plateau that kept higher antibody levels up to 270 dpi. Although PCR-reactivity has no positivity after 4 months post-infection in the mouse model, antibodies are retained for a much longer period (more than 9 months post-infection).

### Preliminary screening of *B. microti* antigens

**Fig. 2:**
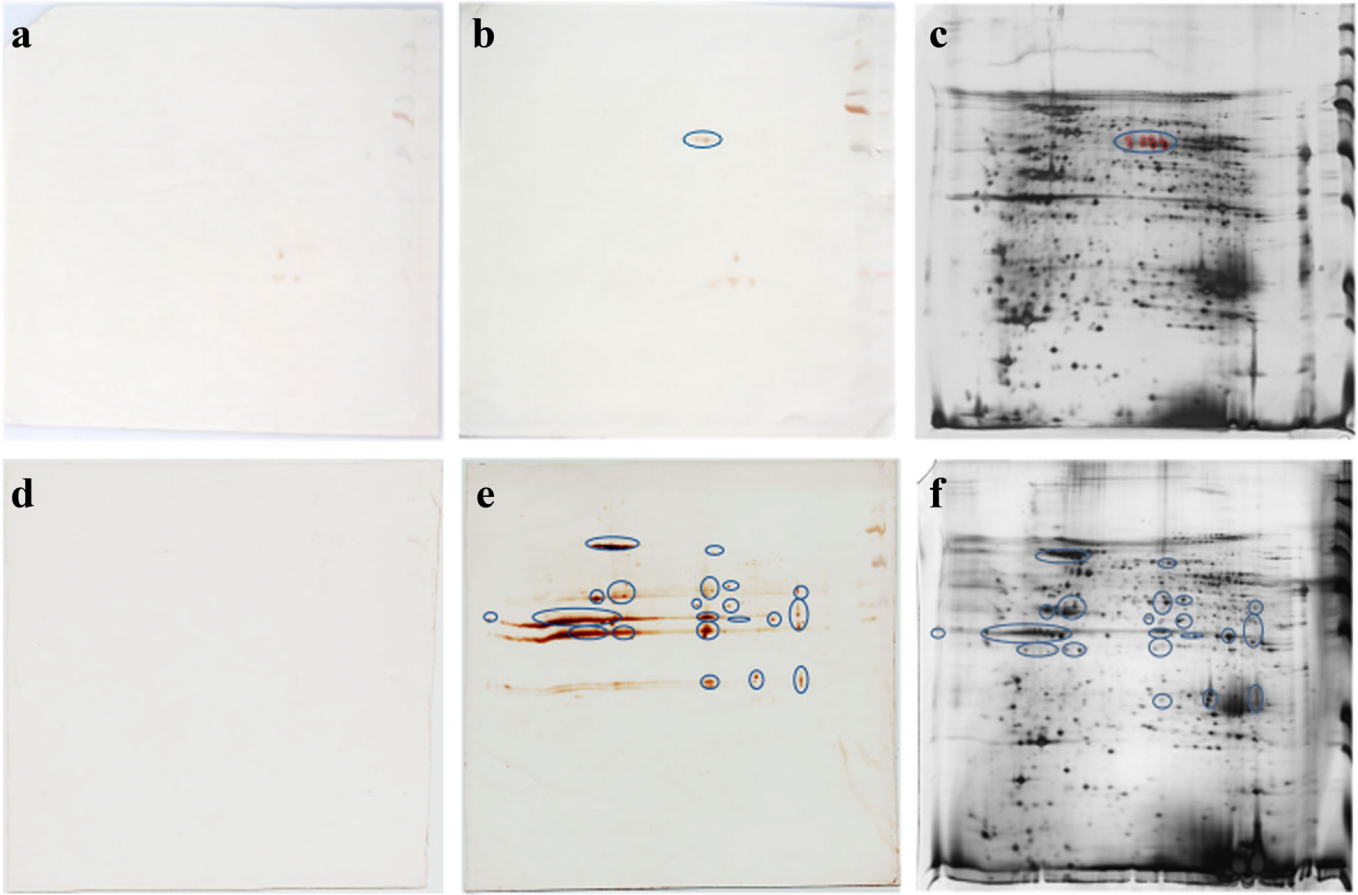
Two-dimensional western blotting. Immune blot patterns of *B. microti* crude proteins were recognized by 7 dpi mice plasma and 30 dpi mice plasma. **a** and **d** provide the results of two-dimensional western blotting (2D-western blotting) incubated by normal mice plasma; **b** and **e** show hybridized membranes incubated by 7 and 30 dpi mice plasma, respectively; **c** and **f** represent the images of silver gel to identify of the crude proteins. Blue circles represent the different areas compared with negative control for *B. microti* crude proteins

To identify potential antigens, the crude *B. microti* proteins were separated on 2-DE gels (Fig. 2c, f). Each sample was subjected to triplicate runs. Upon comparison with immune blots of normal plasma (Fig. 2a, d), 4 and 51 reactive spots were displayed on the membranes hybridized with the 7 dpi and 30 dpi mice, respectively (Fig. 2b, e). In total, 55 localized spots that corresponded to antigenic proteins in 2-DE gels were excised from the silver gels (Fig. 2c, f), and were further analyzed by LC/MS-MS and BLASTp.

In total, 8 proteins were identified by incubating with the plasma of 7 dpi mice. Analyzed with *B. microti* strain RI database by BLASTn, the 8 proteins were peptide alpha-N-acetyltransferase, hypothetical protein and 6 unnamed proteins (Additional file 2: Table S2). Seventy-nine *B. microti* proteins were confirmed by hybridizing 30 dpi mice plasma, including 45 unnamed proteins, eleven hypothetical proteins, seven heat-shock protein 70s, four conserved and unknown function of *Plasmodium* proteins, and some other miscellaneous reported proteins (Additional file 2: Table S2).

### PCR amplification of fragments and In-Fusion cloning

From a total of 128 *B. microti* gene fragments, 113 fragments were successfully amplified (88.3%) (Table 2). The cloning results showed that 109 gene fragments were successfully cloned (96.4%), including all the gene fragments identified by 7 dpi plasma samples (Table 2).

**Table 2:** High-throughput cloning and expression of *B. microti* gene fragments

	*B. microti* gene fragments	PCR amplification (%)	In-fusion cloning (%)	Expressed by the WGCF (%)
Bm2D-30 dpi	119	104 (87.4)	100 (96.2)	80 (80.0)
Bm2D-7 dpi	9	9 (100)	9 (100)	7 (77.8)

*Abbreviation*: *dpi* days post-infection

**Fig. 3:**
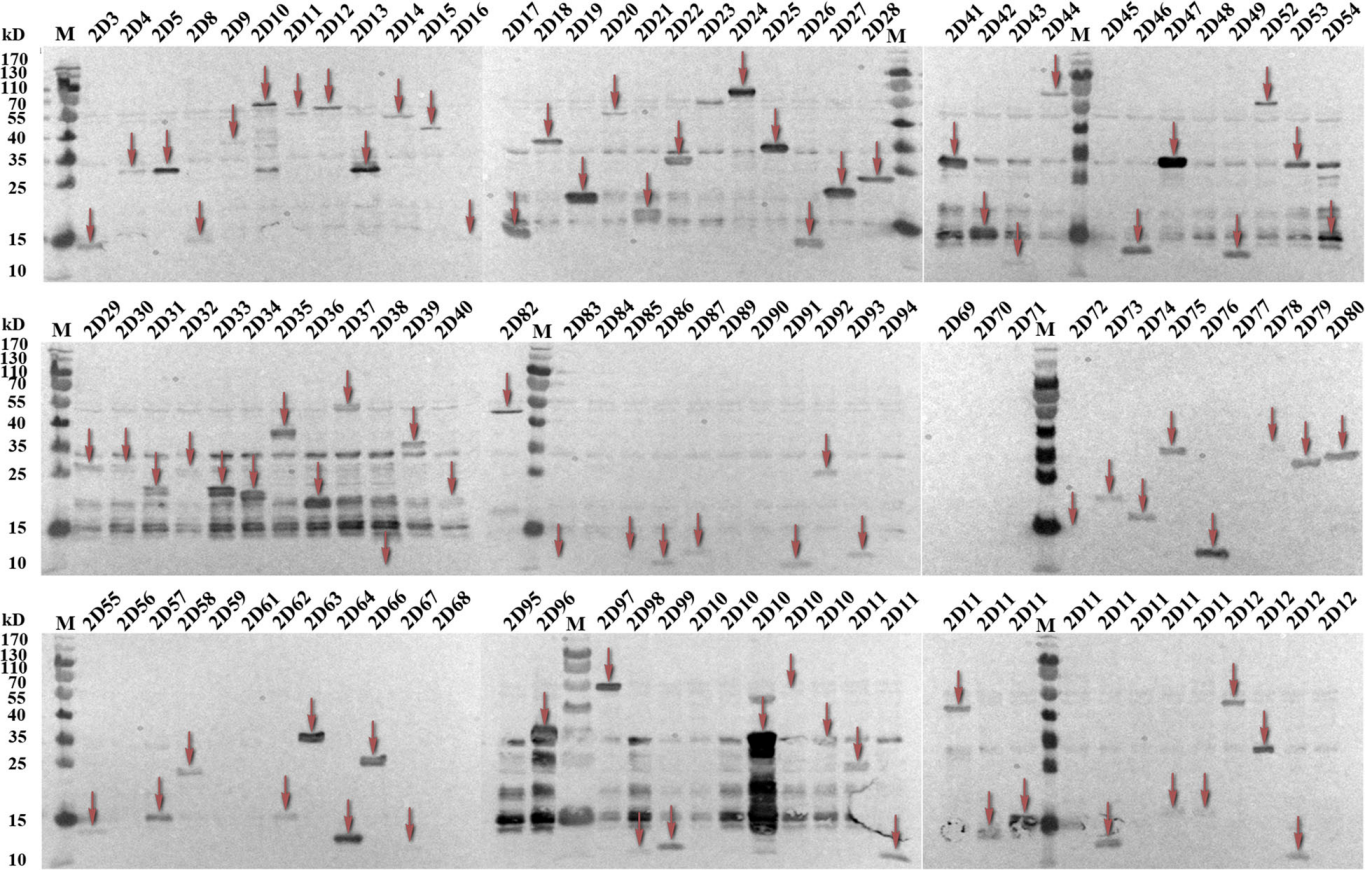
Western blot analysis of the expression levels of *B. microti* proteins. M represents protein size marker, Target proteins are marked with red arrows

Of the 109 fragments, 87 (79.8%) yielded protein products detected by western blot (Table 2, Fig. 3). However, few proteins migrated differently compared with the expected, as which has been reported for a variety of proteins expressed by the WGCF system [46].

### Acquiring immunoreactivity profiles

**Fig. 4:**
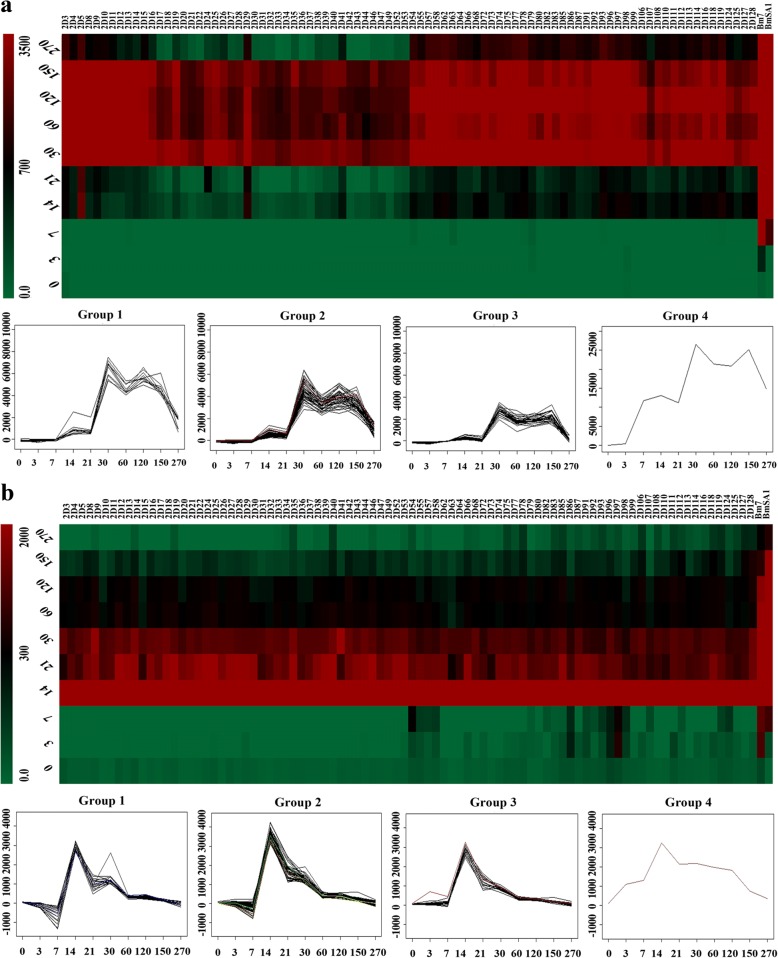
Immunoreactivity patterns of *B. microti* proteins. **a** and **b** show that a total 87 antigens exhibit IgG and IgM, respectively, antibody responses to different period plasma samples isolated by mice inoculation (0, 3, 7, 14, 21, 30, 60, 120, 150 and 270 dpi). Immunoreactivity profiles were clustered according to fluorescence intensity values (M-values). On the heatmap, the scale is from 0 to 3500 and 0 to 2000 in IgG and IgM antibody responses, respectively

The protein array with eighty-seven recombinant proteins were produced and probed with the pooled plasma of ten mice across ten different time points (0, 3, 7, 14, 21, 30, 60, 120, 150 and 270 dpi) to detect their immunoreactivity with IgG and IgM, respectively (Additional file 6: Figure S1). The different immunoreactivity profiles with IgG and IgM were presented as heatmaps Fig. 4a and b, respectively, for interpretation.

Examining IgG antibody responses, it was shown that plasma from 0, 3, 7 and 270 dpi subjects showed low reactivity against all WGCF expressed proteins (Additional file 6: Figure S1A: a-c, j), whereas plasma from 14 to 150 dpi subjects showed clear reactivity (Additional file 6: Figure S1A: d-i). The heatmap shows four different patterns, Group 1 (11 antigens), Group 2 (47 antigens), Group 3 (29 antigens) and Group 4 (only one antigen, which was purified as a recombinant protein expressed in *E. coli*), were clustered showed in Fig. 4a and Additional file 7: Table S6.

Four patterns presented respectively very high, high, moderate and low immunoreactivity. According to their expression patterns, as described below, five antigens were selected for the further evaluation, including antigens 2D5 (AAO18095.1/ BMR1_02g04275-t32_1), 2D29 (CCF74204.1/ BMR1_03g00005-t32_1), 2D41 (CCF75408.1/ BmR1_04g06050-t32_1) and Bm7 (CCF73510.1) from Group 1, Group 2, Group 3 and Group 4 with high reaction from 14 dpi to 270 dpi, as well as antigen 2D33 (CCF74637.1/ BMR1_03g02171-t32_1) from Group 3 with lower reactivity after 2 months. However, with IgM antibody responses, most proteins showed significant immunoreactivity to the plasma of 14 to 30 dpi (Additional file 6: Figure S1B: d-f), and lower reactivity before 7 dpi and after 30 dpi (Additional file 6: Figure S1B: a-c, g-j). Likewise, four different patterns, Group 1 (19 proteins), Group 2 (50 proteins), Group 3 (18 proteins) and Group 4 (one protein), were clustered showed in Fig. 4b and Additional file 7: Table S6. The first three groups did not present significant differences, only one antigen, 2D97 (CCF75281.1/BmR1_04g05415-t32_1) from Group 3, showed IgM immunoreactivity during 3 dpi and 7 dpi (Fig. 4b, Additional file 6: Figure S1), which may be a possible candidate for early detection. These proteins are also expected as *B. microti* vaccine candidates and diagnosed antigens.

### Evaluation of recombinant proteins as diagnostic and vaccine antigens

rBm2D33 and rBm2D41 were expressed in *Escherichia coli* as soluble proteins and rBm2D97 and rBm7 were produced as inclusion bodies (Additional file 8: Figure S2) with the molecular weights of 42 KDa, 39 KDa, 38 KDa and 24 KDa, respectively.

**Fig. 5:**
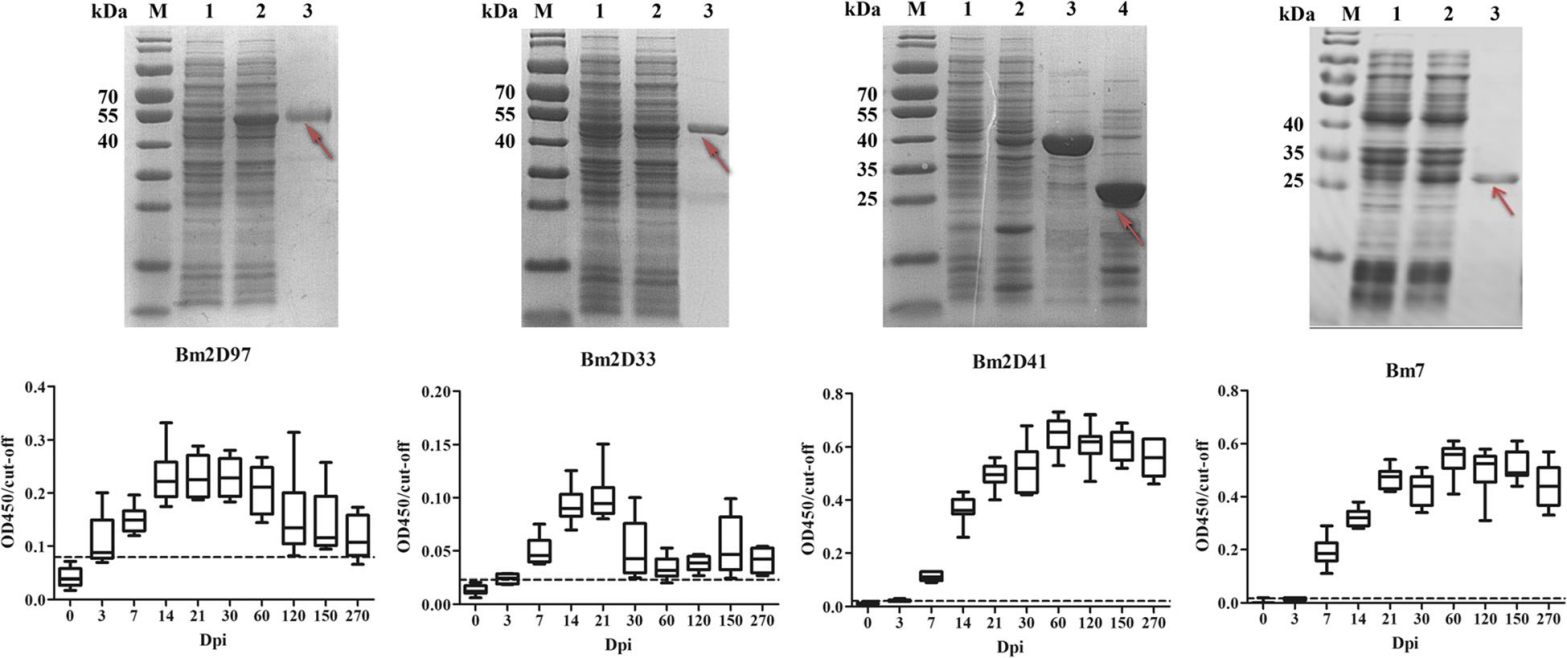
Expression, purification and evaluation of a specific antibody against the recombinant proteins. Lane M: protein size marker; Lane 1: recombinant clone before induction; Lane 2: recombinant clone after induction; Lane 3: purified recombinant protein. Recombinant proteins were evaluated using different periods plasma samples from mice inoculated *B. microti* (0, 3, 7, 14, 21, 30, 60, 120, 150 and 270 dpi) by ELISA

**Fig. 6:**
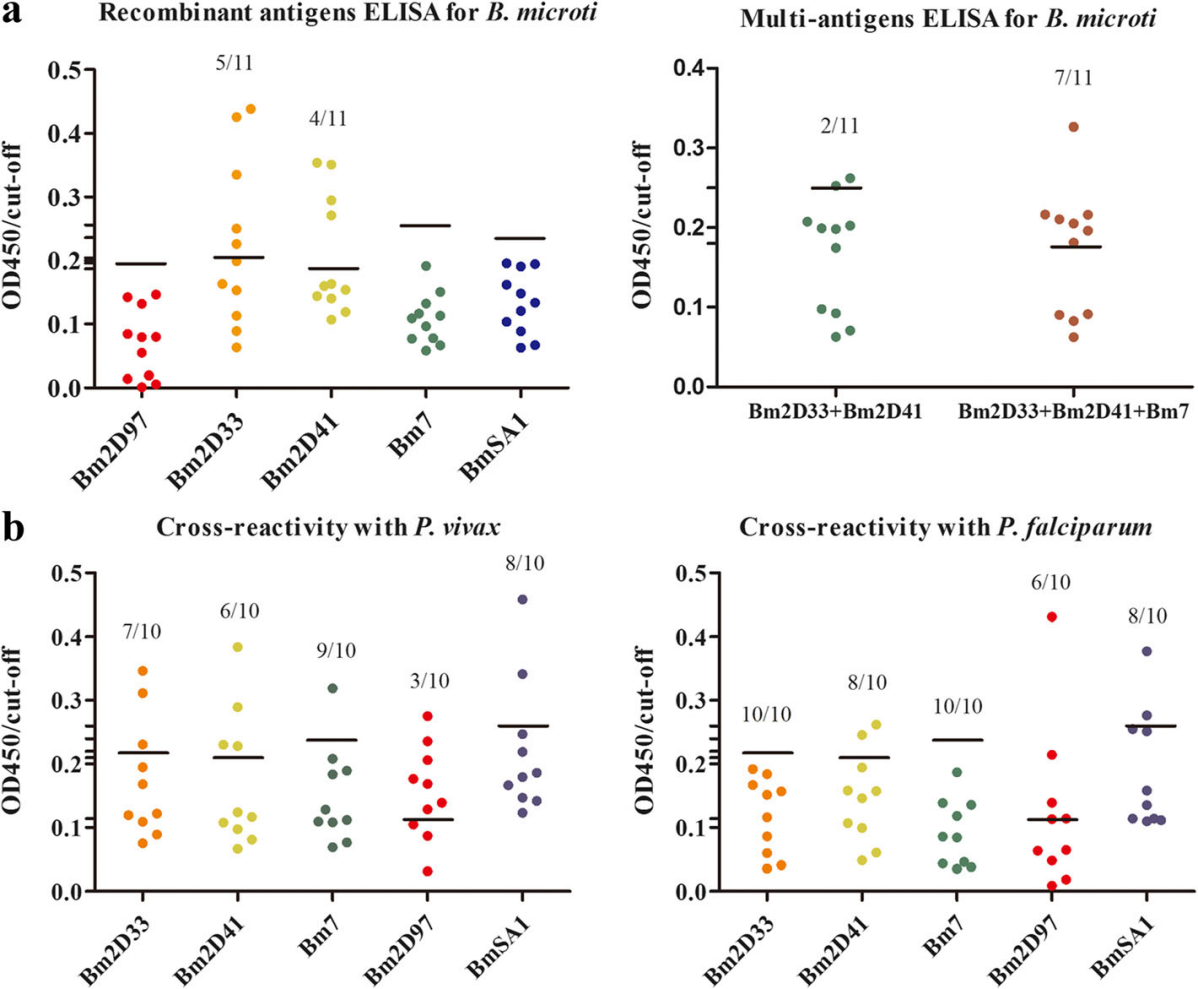
Comparison of the sensitivity and specificity of *B. microti* recombinant antigens. **a** ELISA reactivity of *B. microti* recombinant antigens and multi-antigens to babesiosis patient plasma (*n* = 8). **b** ELISA reactivity of *B. microti* recombinant antigens to *P. vivax* and *P. falciparum* patient plasma (*n* = 10)

The immunoreactivity between the purified antigens and the different time periods of plasma samples from mice inoculated *B. microti* (0, 3, 7, 14, 21, 30, 60, 120, 150 and 270 dpi) showed similar trends when compared with heatmap analysis of protein arrays (Figs. 4, 5). rBm2D33 and rBm2D41 showed better characterization of *B. microti* diagnostic protein in ELISA reactivity, which 62.5% (5 of 8 tested samples) and 50% (4 of 8 tested samples), respectively, compared to rBmSA1 with no obvious detection (Fig. 6a). The reactivity between the patients’ plasma and the multi-antigens combined with rBm2D33, rBm2D41 and rBm7, showed similar sensitivity to rBm2D33, which show 62.5% of samples testing positive (Fig. 6a).

Furthermore, the specificity of the diagnostic protein markers was evaluated by cross-reactivity to known *Plasmodium* infections. The reaction with 10 vivax malaria plasma samples showed there were 1 (10%), 2 (20%), 3 (30%), 4 (40%) and 7 (70%) diagnosed as being positive by rBm7, rBmSA1, rBm2D33, rBm2D41 and rBm2D97, respectively (Fig. 6b). Furthermore, the reaction with 10 falciparum malaria plasma samples, showed no samples diagnosed as being positive by rBm7 and rBm2D33, and 2 (20%) by rBm2D41and rBmSA1, and 4 (40%) by rBm2D97 diagnosed as being positive (Fig. 6b). Therefore, the sensitivity and cross-reactivity to *Plasmodium* of rBm2D33 was better than other antigens.

Two hundred febrile cases from a malaria endemic area (Tengchong, Yunnan Province) were used to evaluate the infection state of *B. microti*, *P. falciparum* and *P. vivax*. The PCR detection method results showed that there were ten positive cases of *B. microti*, 14 and 4 were positive cases of *P. vivax* and *P. falciparum*, respectively, including one co-infection case of *P. vivax* and *P. falciparum,* but no co-infection of *Plasmodium* or *B. microti* case was found. These 200 plasma samples were analyzed by ELISA using all selected *B. microti* antigens (Additional file 9: Figure S3), and samples identified as positive are displayed in Table 3. Finally, the results of evaluating febrile cases are displayed in heatmap including nested-PCR and recombinant proteins by ELISA (Additional file 10: Figure S4). Of the 200 febrile cases examined, 32 and 34 were detected as being positive by rBm2D41 and rBm7 which included 1and 2 PCR positive cases of *B. microti* and 7 PCR positive cases of *Plasmodium*, respectively. Thirty-two were detected as being positive by rBm2D33 which included 2 PCR-positive cases of *B. microti* and 9 PCR-positive cases of *Plasmodium*. Twenty-seven were detected as being positive by rBm2D97 which included 1 PCR positive case of *B. microti* and 10 PCR-positive cases of *Plasmodium*. rBmSA1 and crude *B. microti* proteins were used as controls. Thirty-eight and fifty-eight were detected as being positive, including 1 and 3 PCR-positive cases of *B. microti* and 14 and 9 PCR-positive cases of *Plasmodium.* Thus, more antibody-positive and PCR-negative cases were present in the examined cohort.

**Table 3:** Samples positive for *B. microti* in febrile cases

Detection method		Recombinant proteins as diagnostic antigens					
**PCR**	ELISA	Crude protein	rBmSA1	rBm2D41	rBm7	rBm2D97	rBm2D33
+	–	7	9	9	8	9	8
+	+	3	1	1	2	1	2
–	+	55	37	31	32	26	30

### Vaccination and challenge infection

**Fig. 7:**
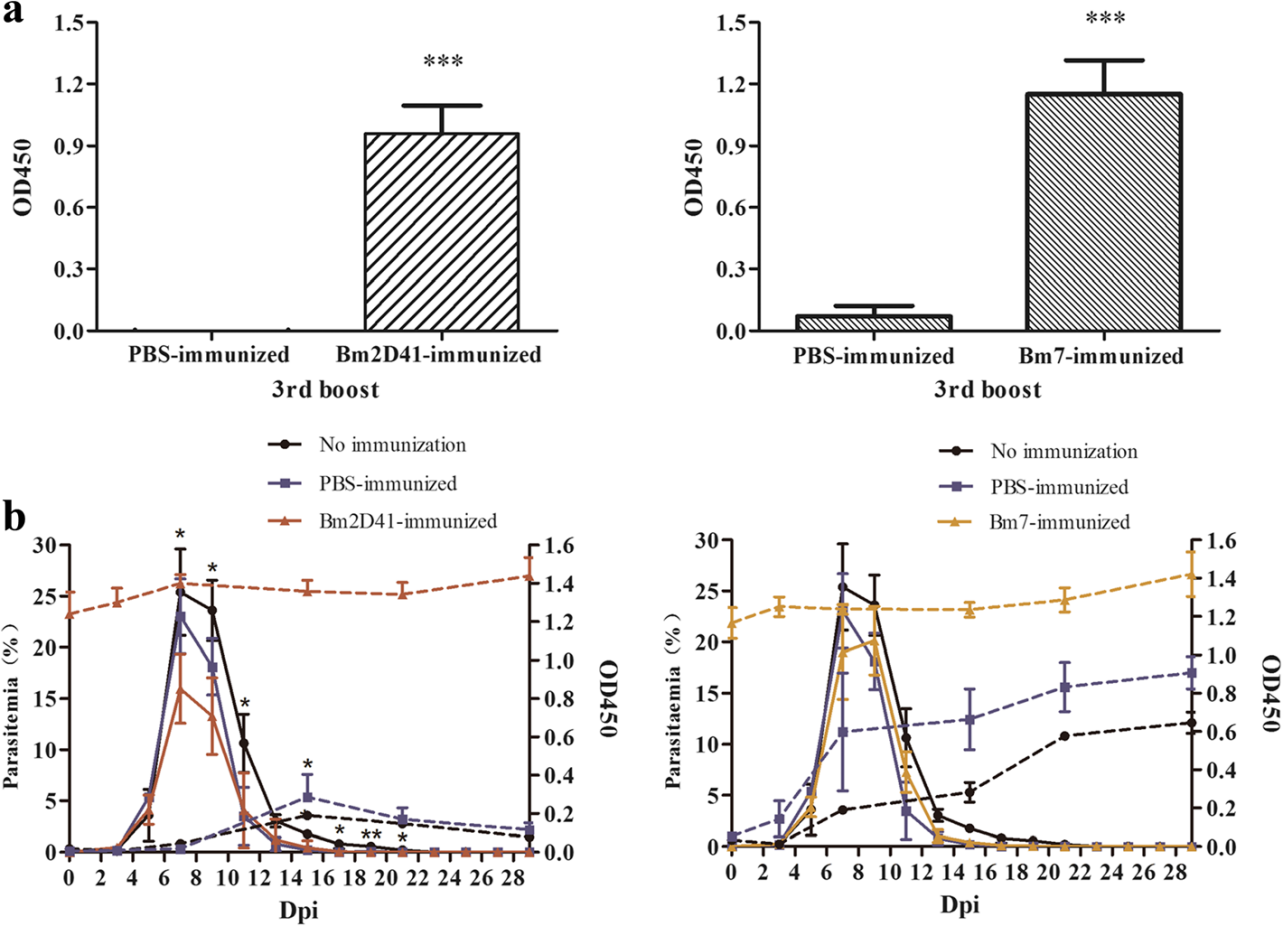
The evaluation and effect of immunization with Bm2D41 and Bm7. **a** The levels of IgG immunized with Bm2D41 and Bm7 in BALB/c mice. **b** The parasitaemia and antibody levels of BABL/c mice after challenge infection with *B. microti*. Asterisks indicate statistically significant differences [*P < 0.05, **P < 0.005, ***P < 0.0001 (compared to either the PBS-immunized or non-immunized BABL/c mice)]. The solid and dashed lines denote parasitaemia and antibody titter, respectively

The antigens with high immunoreactivity were considered to be of great potential for use in a vaccine. Based on the preliminary screening and further immunoreaction evaluation results, we found rBm7 and rBm2D41could induce higher antibody expression. Thus, the parasitaemia and antibody levels of mice immunized with Bm2D41 and Bm7 mice were evaluated. After the 3rd booster for the immunizations, the specific antibody levels in mice plasma had significantly increased (*t*_(4)_ = 15.64, *P* = 2.9 × 10^-7^ and *t*_(4)_ = 13.90, *P* = 6.5 × 10^-7^, respectively) (Fig. 7a) and all mice were challenged with 1 × 10^7^ *B. microti*-infected erythrocytes. In the Bm2D41-immunized group, the parasitaemia were clearly lower than those of the control mice that received either PBS or no immunization during from days 7–21 (*F*_(2, 7)_ = 4.79–4.78, *P* = 0.0031–0.049), except for Day 13 (*F*_(2, 7)_ = 2.255, *P* = 0.1755) (Fig. 7b). Peak parasitaemia appeared at 7 days after the challenge infection and reached an average of 25.4% and 23.0% in the control group of immunization with PBS-immunized and that of no immunization group, respectively. However, compared with control group, the Bm2D-41 immunized group showed the lower parasitaemia peak of 15.9%, while the parasitaemia of the Bm7 immunized group did not decline significantly. Freund’s adjuvant itself could affect the host and cause a certain inhibition of parasitaemia. Parasites had not been completely eliminated by day 30 post-challenge in all groups tested. After the final booster immunization, the antibody titers were maintained at a high level up to the chronic stage of infection in both candidate vaccines (Fig. 7).

## Discussion

Different clinical presentations have been reported in babesiosis, including asymptomatic infection, malaria-like complications or even death [47]. Due to its broad clinical spectrum, misdiagnoses and missed diagnoses are often encountered. Currently, babesiosis is identified by a direct-smear evaluation, antibody tests, PCR assays and animal sub-inoculation [7, 48]. Microscopic examination with a Giemsa-stained blood smear was considered to be the gold-standard for a babesiosis diagnosis. However, it is difficult to discriminate *B. microti* in blood smears, because of low parasitaemia levels and their morphological similarity to *Plasmodium* spp., which may result in inaccurate diagnosis. Comparatively, PCR diagnosis methods while more sensitive and specific, are still not accurate enough for the diagnosis of low parasitaemias or samples during window infection periods. Antibody diagnosis was highly sensitive and suitable for low parasitaemias detection. However, it was still not effective in detecting the infection during the window period for antibody detection. Furthermore, false positives are often eventually identified later in situations where the antibody is present even after the clearance of *B. microti* [7, 47–49].

In this study, the percentage of parasitized erythrocytes, nucleic acid and antibody levels, as well as their correlation were analyzed in the BABL/c mouse model. The results indicate that the percentage of parasitized erythrocytes reached the peak after one week post-inoculation, and markedly reduced until the clearance of the parasite. The detected percentages of parasitized erythrocytes in this study was consistent with previously reported investigations, both in animal models and in the clinic [9, 14, 17, 19]. The window period for antibody analysis is 0 to 7 dpi. With the gradual reduction of the parasitaemia percentage, IgG increased and reached a peak after 30 days, and the high IgG level could be maintained for 9 months. These results indicate that antibody levels could be used as an indicator of *B. microti* infection. In addition, our results show that the specific IgG played important roles in the immune protection in babesiosis.

The current study also showed that PCR analysis was more sensitive than blood smear evaluations and could be complementary to antibody diagnosis during the window period of the infection. However, it was clear that the period for PCR detection was relatively short. A study reporting blood screening in the USA showed that a portion of blood donors could remain antibody-positive for more than one year. Compared to the PCR results, donors with high level of antibodies were more likely to be active or recently infected with *B. microti* than PCR-negative donors with low-titer antibodies [16, 18]. Thus, the antibody diagnosis result was an important parameter for assessment of *B. microti* infection, which was especially effective in diagnosing asymptomatic infections.

In previous research focusing on immune protection mechanisms, IgM was shown as an early response to the *B. microti* infection. Also, IgM levels gradually reached a peak with the increased level of parasitaemia while specific IgG response increased along with the decline of parasitaemia. When IgG reached the peak, parasites were cleared or were present in much lower levels [14]. In our study, the *B. microti* crude antigenic proteins were screened by the plasma collected from the peak of parasitaemia and antibody levels. We compared 87 2D-western positive proteins with the top twenty reactive proteins showed in publication of Silva et al. [22] and 19 *B. microti* GPI proteins showed in publication of Cornillot et al. [21] and found two proteins that were identified in the first comparison, which are 2D5 (AAO18095.1/BMR1_02g04275-t32_1) and 2D13(CCF73151.1/BMR1_01G03465-t32_1). However, none was hit within 19 *B. microti* GPI proteins. We consider the possible reasons maybe related with the different strains and time points used for plasma screening reactive antigen candidates. Furthermore, we also payed attention to BmSA1, which is one of the most expressed proteins, yet was missed in this preliminary screening. However, it was shown that several of the highly antigenic proteins are the most expressed ones [22]. This result indicated that the 2D-western screening may have missed some proteins when they matched the positive reaction protein based on their position on the two gels. Therefore, we combined the 2D-western and bioinformatics analysis for the preliminary screening. The protein microarray results presented in the heatmap analysis showed the trends of immune reaction detected with recombinant antigens during the different periods of infected BALB/c mice. This showed similar dynamics to that of antibody levels tested by crude *B. microti* proteins. Several antigens presented high immune reactions, including 2D5, 2D29 (truncated expressed 2D5), 2D41 and Bm7. These antigens could be the potential diagnostic biomarkers for *B. microti* infection. It was noteworthy that in the animal models, low levels of parasitized erythrocytes or even the apparent absence of parasites, could still present a challenge or secondary infection of *B. microti*, which may differ to the first initial infection [15, 17]. Thus, the antigens with high immune reactions could be of great potential as targets in a vaccine and aid in asymptomatic infection detection. Several antigens with low immunoreactivity as the parasites disappeared or were cleared were detected in the investigation of the antigen spectra, such as 2D33 and 2D36. These antigens could be used as potential biomarkers for the acute period of infection. Low responses were observed when investigating the immunoreactivity profiles of IgM. In accordance with levels of IgM antibody and parasitaemia, the reaction trends of 2D97 showed an early response to parasite infection, thus indicating its potential as a biomarker for early diagnosis of babesiosis.

As discussed above, hosts with positive antibody reactions could still present asymptomatically, but be infected, and thus should be carefully observed. Biomarker antigens with desirable properties with the necessary characterization have been identified in our study and we anticipate could be used developed to screen *B. microti* infections in the future. Although high efficiency in detection of infection in mice were shown, our antigens showed low efficiency with diagnosed patients or infected individuals. This was possibly because of the variance of infection stage and immune response in different hosts [47]. Compared to the reported diagnostic antigen rBMSA1, the rBm2D33 and the combination of rBm2D33, rBm2D41 and rBm7 showed higher sensitivities.

Babesiosis and malaria are similar in symptoms and immune mechanisms, especially when observing the morphology; the *P. falciparum* also has a ring shape in the erythrocyte, which resembled *B. microti****,*** making it difficult to distinguish [47, 50, 51]. Occasionally, a co-infection of *B. microti* and *Plasmodium* parasites is reported [38], leading to misdiagnoses and missed diagnoses. In this regard, a diagnostic antigen is expected to distinguish the *Plasmodium* and *B. microti* infection effectively. In this study, the diagnostic candidate antigens showed low cross-reaction with *P. falciparum* infections compared with *P. vivax* infections, thus the candidate antigens exhibited advantages in distinguishing these two parasites during the infection stage.

Babesiosis is transmitted through infected blood or blood related products, which severely threatens public health [48]. According to a recent report, 97.3% of the positive blood-donation samples tested in the USA were antibody positive, among which 80% were PCR negative. Meanwhile, evidence already indicates the risks in blood transmission, even in the PCR negative samples [18]. In regards to the recent babesiosis cases reported in China [38, 52, 53], two hundred microscopically negative samples of blood cells and plasma were collected in a malaria epidemic area in Tengchong, Yunnan Province, for further tests and screening in our study. The detection of these samples was carried out using the potential antigen biomarkers obtained in this study, crude *B. microti* proteins and PCR, respectively. As a result, the positive rates were 13.5–19.0%, 27.5% and 5%, respectively. PCR negative rates were 72.0–78.0% for potential biomarker antigen positive samples, and 84.6% for crude *B. microti* protein positive samples. The results further highlighted the risks of babesiosis spreading due to the missed diagnoses using only PCR methods.

The potential of the obtained antigens as a possible vaccine was also evaluated in our study. In the immune protection experiment, rBm2D41 reduced the peak value by 37.4%, thus provided an effective protection to the *B. microti* infection, and also prevented serious hemolysis symptoms. The preliminary tests indicated that the potential antigen biomarker also possessed a potential vaccine property, thus our team is encouraged to produce more investigations with biomarker proteins to be screened and discovered. Also, whether the risk of infection by blood transfusion or tick bite could be lowered by the vaccination was also worth investigating.

## Conclusions

The diagnosis technologies for babesiosis, including microscopic examination, PCR assays and antibody tests represent the infection at different stages. This study especially pinpointed the importance of antibody detection. The screened biomarkers for the disease progression during babesiosis infection in this work, provides useful information for the diagnosis and vaccine development for this serious risk in public health.

## Additional files

Additional file 1: **Table S1.** The samples information of patients with babesiosis. (DOCX 14 kb)

Additional file 2: **Table S2.** Identities of *B. microti* crude antigenic proteins recognized by 7 dpi and 30 dpi plasma samples. (DOCX 25 kb)

Additional file 3: **Table S3.** The sequence information of *B. microti* gene fragments and primers of in-fusion clone. (DOCX 51 kb)

Additional file 4: **Table S4.** The primer sequences of *B. microti* genes in prokaryotic cloning and expression. (DOCX 14 kb)

Additional file 5: **Table S5.** The primer sequences of *P. vivax* and *P. falciparum*. (DOCX 14 kb)

Additional file 6: **Figure S1.** Antibody profiling of *B. microti* proteins by protein arrays. Crude *B. microti* proteins (2D3-2D54, 2D55-2D128) react with plasma samples across ten different time points (a-j: 0, 3, 7, 14, 21, 30, 60, 120, 150 and 270 dpi). The reactions were detected with anti-mouse IgG (A) and anti-mouse IgM (B). Control reactions of wheat germ lysate that lacked vector templates (white box) and reactions of purified recombinant proteins (red box) served as negative and positive controls. Well characterized reactions with target proteins are marked with orange boxes. (TIF 3404 kb)

Additional file 7: **Table S6.** *Babesia microti* proteins were grouped by immunoreactivity patterns. (DOCX 17 kb)

Additional file 8: **Figure S2.** Soluble expression analysis of rBm2D97, rBm2D33, rBm2D41 and rBm7. M: MW markers; Lane 1: pre-induction extraction of whole-cell protein; Lane 2: pre-induction supernatant; Lane 3: pre-induction precipitation; Lane 4: extraction of whole-cell proteinpost IPTG induction; Lane 5: supernatant post IPTG induction; Lane 6: precipitation post IPTG induction. Target proteins are marked with red arrow. (TIFF 1265 kb)

Additional file 9: **Figure S3.** Two hundred febrile cases evaluated by crude *B.microti* proteins and recombinant antigens. (TIFF 417 kb)

Additional file 10: **Figure S4.** Evaluating febrile cases using nested-PCR and recombinant proteins by ELISA. (TIF 6386 kb)

## Abbreviations

2-DE: Two-dimensional electrophoresis
ANOVA: One-way analysis of variance
CAMS: Chinese Academy of Medical Sciences
China CDC: Chinese Center for Disease Control and Prevention
DAB: 3, 3'-Diaminobenzidine
dpi: Days post-infection
ELISA: Enzyme-linked immunosorbent assay
FDA: Food and Drug Administration
GPI: Glycosyl-phosphatidyl inositol
HCA: Hierarchical clustering analysis
IgG: Immunoglobulin G
IgM: Immunoglobulin M
LC/MS: Liquid chromatography-mass spectrometry
MeV: Multi-array experiment viewer
NIPD: National Institute of Parasitic Diseases
OD: Optical density
PCR: Polymerase-chain-reaction
PVDF: Polyvinylidene fluoride
SD: Standard deviation
SDS-PAGE: Sodium dodecyl sulfate polyacrylamide gel electrophoresis
TTB: Transfusion-transmitted babesiosis
WGCF: Wheat germ cell-free
