# Supplementary material for: Screening for biomarkers reflecting the progression of Babesia microti infection
Source: Parasit Vectors. 2018 Jul 3;11:379. doi: 10.1186/s13071-018-2951-0 (PMC6029176; doi:10.1186/s13071-018-2951-0)
Supplement: Supplementary file 1 — Table S1. The samples information of patients with babesiosis. (DOCX 14 kb) [file 13071_2018_2951_MOESM1_ESM.docx]

**Additional file 1: Table S1. The samples information of patients with babesiosis**

| Case number | Location of collection | Disease | Pathogens | Diagnosis | | | | Remarks |
| --- | --- | --- | --- | --- | --- | --- | --- | --- |
|  |  |  |  | Symptoms | Etiological techniques | Immunology | Mo1ecular bio1ogical techniques(18S rRNA) |  |
| 1 | Guangdong | Babesiosis | *B.*  *microti*-like | Fever, hepatosplenomegaly and myeloma | Intraerythrocytic trophozoite-like organisms | ND* | 431bp | *Babesia* sp |
| 2 | Shanghai | Babesiosis | *B. microti*-like | Fever | Intraerythrocytic trophozoite-like organisms | ND | 431bp | *Babesia* sp |
| 3 | Fujian | Babesiosis | *B. microti*-like | Fever, hepatosplenomegaly and lymphoma | No | ND | 407bp and 1620bp with full length | 87% homology with *B. microti* |
| 4 | Henan | Babesiosis | *B. microti*-like | Fever, hepatosplenomegaly and lymphoma | No | ND | 427bp | *Babesia* sp |
| 5 | Xinjiang | Babesiosis | *B. microti*-like | Fever and hepatosplenomegaly | No | ND | 431bp | *Babesia* sp |
| 6 | Shanghai | Babesiosis | *B. microti*-like | Fever and hepatosplenomegaly | Intraerythrocytic trophozoite-like organisms | ND | 431bp and 1620bp with full length | 85% homology with *B. microti* |
| 7 | Fujian | Babesiosis | *B. microti*-like | Fever and hepatosplenomegaly | Intraerythrocytic dots of chromatin and trophozoite-like organisms | Dot-ELISA positive | 431bp and 1537bp with full length | 87% homology with *B. microti* |
| 8 | Shanghai | Babesiosis | *B. microti*-like | Fever | Intraerythrocytic dots of chromatin organisms | ND | 431bp and 1537bp with full length | 88% homology with *B. microti* |

*ND. not detectable.
