# Supplementary material for: Screening for biomarkers reflecting the progression of Babesia microti infection
Source: Parasit Vectors. 2018 Jul 3;11:379. doi: 10.1186/s13071-018-2951-0 (PMC6029176; doi:10.1186/s13071-018-2951-0)
Supplement: Supplementary file 2 — Table S2. Identities of B. microti crude antigenic proteins recognized by 7 dpi and 30 dpi plasma samples. (DOCX 25 kb) [file 13071_2018_2951_MOESM2_ESM.docx]

**Additional file 2: Table S2. Identities of *B. microti* crude antigenic proteins recognized by 7 dpi and 30 dpi serum samples**

| **Blotting** | **PMID** | | **Protein Name** |
| --- | --- | --- | --- |
| Bm2D-7 dpi | CCF74602.1 | BMR1_03g02000-t32_1 | unnamed protein product [*Babesia microti* strain RI] |
|  | CCF74416.1 | BMR1_03g01070-t32_1 | unnamed protein product [*Babesia microti* strain RI] |
|  | CCF72650.1 | BMR1_01G00940-t32_1 | unnamed protein product [*Babesia microti* strain RI] |
|  | CCF75986.1 | -- | peptide alpha-N-acetyltransferase [Babesia microti strain RI] |
|  | CCF75994.1 | BmR1_04g09095-t32_1 | unnamed protein product [*Babesia microti* strain RI] |
|  | CCF74336.1 | BMR1_03g00665-t32_1 | unnamed protein product [*Babesia microti* strain RI] |
|  | CCF74285.1 | BMR1_03g00410-t32_1 | unnamed protein product [*Babesia microti* strain RI] |
|  | CCF73918.1 | BMR1_02g03840-t32_1 | hypothetical protein [*Babesia microti* strain RI] |
| Bm2D-30 dpi | CCF75793.1 | BmR1_04g08040-t32_1, | molecular chaperone DnaK [*Babesia microti* strain RI] |
|  | CCF72966.1 | BMR1_01G02545-t32_1 | heat shock 70kDa protein 1/8 [*Babesia microti* strain RI] |
|  | BAF02622.1 | BMR1_01G02545-t32_1 | heat shock protein 70 [*Babesia microti* strain RI] |
|  | BAF02621.1 | BMR1_01G02545-t32_1 | heat shock protein 70 [*Babesia microti* strain RI] |
|  | AAC47456.1 | BMR1_01G02545-t32_1 | heat shock protein 70 [*Babesia microti* strain RI] |
|  | BAF02621.1 | BMR1_01G02545-t32_1 | heat shock protein 70 [*Babesia microti* strain RI] |
|  | CCF72966.1 | BMR1_01G02545-t32_1 | HSPA1_8, heat shock 70kDa protein 1/8[*Babesia microti* strain RI] |
|  | CCF75408.1 | BmR1_04g06050-t32_1 | heat shock 70kDa protein 5 [*Babesia microti* strain RI] |
|  | BAH28858.1 | BmR1_04g06050-t32_1 | glucose regulated stress protein, partial [*Babesia microti* strain RI] |
|  | CCF75640.1 | BmR1_04g07221-t32_1 | unnamed protein product [*Babesia microti* strain RI] |
|  | CCF73712.1 | BMR1_02g02790-t32_1 | large subunit ribosomal protein L4e [Babesia microti strain RI] |
|  | CCF74956.1 | BMR1_03g03765-t32_1 | unnamed protein product [*Babesia microti* strain RI] |
|  | CCF75517.1 | BmR1_04g06605-t32_1 | unnamed protein product [*Babesia microti* strain RI] |
|  | CCF74637.1 | BMR1_03g02171-t32_1 | unnamed protein product [*Babesia microti* strain RI] |
|  | CCF73790.1 | BMR1_02g03185-t32_1 | unnamed protein product [*Babesia microti* strain RI] |
|  | CCF72517.1 | BMR1_01G00234-t32_1,  BMR1_01G00230-t32_1 | glyceraldehyde 3-phosphate dehydrogenase, GAPDH [*Babesia microti* strain RI] |
|  | CCF72520.1 | BMR1_01G00234-t32_1, BMR1_01G00230-t32_1 | glyceraldehyde 3-phosphate dehydrogenase, GAPDH [*Babesia microti* strain RI] |
|  | LN871598.1 | BMR1_03g00685-t32_1 | unnamed protein product [*Babesia microti* strain RI] |
|  | CCF73151.1 | BMR1_01G03465-t32_1 | conserved Plasmodium protein, unknown function [*Babesia microti* strain RI] |
|  | CCF75386.1 | BmR1_04g05940-t32_1 | large subunit ribosomal protein LP1 [*Babesia microti* strain RI] |
|  | CCF74238.1 | BMR1_03g00175-t32_1 | unnamed protein product [*Babesia microti* strain RI] |
|  | CCF76124.1 | BmR1_04g09785-t32_1 | unnamed protein product [*Babesia microti* strain RI] |
|  | CCF73865.1 | BMR1_02g03565-t32_1 | RING zinc finger protein, putative [*Babesia microti* strain RI] |
|  | CCF73504.1 | -- | hypothetical protein [*Babesia microti* strain RI] |
|  | CCF72674.1 | BMR1_01G01040-t32_1 | CCR4-NOT transcription complex subunit 9 [*Babesia microti* strain RI] |
|  | CCF75956.1 | BmR1_04g08895-t32_1 | hypothetical protein [*Babesia microti* strain RI] |
|  | CCF73397.1 | BMR1_02g01170-t32_1 | unnamed protein product [*Babesia microti* strain RI] |
|  | CCF72475.1 | BMR1_01G00004-t32_1 | BMN1 family, Pseudo gene [*Babesia microti* strain RI] |
|  | CCF73474.1 | BMR1_02g01565-t32_1 | conserved Plasmodium protein, unknown function  [*Babesia microti* strain RI] |
|  | CCF74240.1 | BMR1_03g00185-t32_1 | unnamed protein product [*Babesia microti* strain RI] |
|  | CCF74836.1 | BMR1_03g03165-t32_1 | unnamed protein product [*Babesia microti* strain RI] |
|  | CCF75326.1 | BmR1_04g05640-t32_1 | PWP1, periodic tryptophan protein 1 [*Babesia microti* strain RI] |
|  | CCF72898.1 | BMR1_01G02191-t32_1 | unnamed protein product [*Babesia microti* strain RI] |
|  | CCF73248.1 | BMR1_02g00410-t32_1 | 20S proteasome subunit beta 4 [*Babesia microti* strain RI] |
|  | CCF72846.1 | BMR1_01G01931-t32_1 | unnamed protein product [*Babesia microti* strain RI] |
|  | CCF75859.1 | BmR1_04g08390-t32_1 | unnamed protein product [*Babesia microti* strain RI] |
|  | BAB83929.1 | BMR1_03g04240-t32_1 | T-complex protein 1 [*Babesia microti* strain RI] |
|  | BAH22737.1 | BMR1_03g04240-t32_1 | eta subunit of chaperonin containing t-complex polypeptide 1 [*Babesia microti* strain RI] |
|  | CCF74742.1 | BMR1_03g02695-t32_1 | unnamed protein product [*Babesia microti* strain RI] |
|  | CCF74204.1 | BMR1_03g00005-t32_1 | unnamed protein product, partial [*Babesia microti* strain RI] |
|  | AAO18095.1 | BMR1_02g04275-t32_1 | N1-21 subtype b protein [*Babesia microti* strain RI] |
|  | CCF73594.1 | BMR1_02g02180-t32_1 | hypothetical protein [*Babesia microti* strain RI] |
|  | CCF76062.1 | BmR1_04g09455-t32_1 | tubulin alpha [*Babesia microti* strain RI] |
|  | CCF75929.1 | BmR1_04g08755-t32_1 | DNA-directed RNA Polymerase II subunit L [*Babesia microti* strain RI] |
|  | CCF74546.1 | BMR1_03g01720-t32_1 | unnamed protein product [*Babesia microti* strain RI] |
|  | CCF72691.1 | BMR1_01G01121-t32_1 | unnamed protein product [*Babesia microti* strain RI] |
|  | CCF72679.1 | BMR1_01G01065-t32_1 | unnamed protein product [*Babesia microti* strain RI] |
|  | CCF75648.1 | BmR1_04g07270-t32_1 | hypothetical protein [*Babesia microti* strain RI] |
|  | CCF73033.1 | BMR1_01G02895-t32_1 | hypothetical protein [*Babesia microti* strain RI] |
|  | CCF75281.1 | BmR1_04g05415-t32_1 | conserved Plasmodium protein, unknown function  [*Babesia microti* strain RI] |
|  | CCF75011.1 | BMR1_03g04040-t32_1 | unnamed protein product [*Babesia microti* strain RI] |
|  | CCF74546.1 | BMR1_03g01720-t32_1 | unnamed protein product [*Babesia microti* strain RI] |
|  | CCF75913.1 | BmR1_04g08680-t32_1 | hypothetical protein [*Babesia microti* strain RI] |
|  | CCF74961.1 | BMR1_03g03790-t32_1 | unnamed protein product [*Babesia microti* strain RI] |
|  | CCF73488.1 | BMR1_02g01640-t32_1 | unnamed protein product [*Babesia microti* strain RI] |
|  | CCF76134.1 | BmR1_04g09835-t32_1 | hypothetical protein [*Babesia microti* strain RI] |
|  | CCF75944.1 | BmR1_04g08832-t32_1 | unnamed protein product [*Babesia microti* strain RI] |
|  | CCF73197.1 | BMR1_02g00155-t32_1 | unnamed protein product [*Babesia microti* strain RI] |
|  | CCF73838.1 | BMR1_02g03430-t32_1 | unnamed protein product [*Babesia microti* strain RI] |
|  | CCF73803.1 | BMR1_02g03250-t32_1 | hypothetical protein [*Babesia microti* strain RI] |
|  | CCF75049.1 | BMR1_03g04230-t32_1 | unnamed protein product [*Babesia microti* strain RI] |
|  | CCF73331.1 | BMR1_02g00830-t32_1 | unnamed protein product [*Babesia microti* strain RI] |
|  | CCF73722.1 | BMR1_02g02840-t32_1 | peptidyl-prolyl cis-trans isomerase-like 2  [*Babesia microti* strain RI] |
|  | CCF73602.1 | BMR1_02g02220-t32_1 | conserved Plasmodium protein, unknown function  [*Babesia microti* strain RI] |
|  | CCF75422.1 | BmR1_04g06120-t32_1 | unnamed protein product [*Babesia microti* strain RI] |
|  | CCF75298.1 | BmR1_04g05500-t32_1 | hypothetical protein [*Babesia microti* strain RI] |
|  | CCF75101.1 | BMR1_03g04495-t32_1 | unnamed protein product [*Babesia microti* strain RI] |
|  | CCF73895.1 | BMR1_02g03725-t32_1 | unnamed protein product [*Babesia microti* strain RI] |
|  | CCF72738.1 | BMR1_01G01371-t32_1 | unnamed protein product [*Babesia microti* strain RI] |
|  | CCF75374.1 | BmR1_04g05880-t32_1 | ribonucleoside-diphosphate reductase subunit M1 [EC:1.17.4.1] [*Babesia microti* strain RI] |
|  | CCF74749.1 | BMR1_03g02730-t32_1 | unnamed protein product [*Babesia microti* strain RI] |
|  | CCF74681.1 | BMR1_03g02390-t32_1 | unnamed protein product [*Babesia microti* strain RI] |
|  | CCF72705.1 | BMR1_01G01200-t32_1 | unnamed protein product [*Babesia microti* strain RI] |
|  | CCF73920.1 | BMR1_02g03850-t32_1 | XAP5 circadian clock regulator [*Babesia microti* strain RI] |
|  | CCF72700.1 | BMR1_01G01170-t32_1 | unnamed protein product [*Babesia microti* strain RI] |
|  | CCF73388.1 | BMR1_02g01125-t32_1 | hypothetical protein [*Babesia microti* strain RI] |
|  | CCF76139.1 | BmR1_04g09851-t32_1 | unnamed protein product [*Babesia microti* strain RI] |
|  | CCF73794.1 | BMR1_02g03205-t32_1 | unnamed protein product [*Babesia microti* strain RI] |
|  | CCF73900.1 | BMR1_02g03750-t32_1 | unnamed protein product [*Babesia microti* strain RI] |
