# Supplementary material for: Screening for biomarkers reflecting the progression of Babesia microti infection
Source: Parasit Vectors. 2018 Jul 3;11:379. doi: 10.1186/s13071-018-2951-0 (PMC6029176; doi:10.1186/s13071-018-2951-0)
Supplement: Supplementary file 3 — Table S3. The sequence information of B. microti gene fragments and primers of in-fusion clone. (DOCX 51 kb) [file 13071_2018_2951_MOESM3_ESM.docx]

| **Additional file 3: Table S3. The sequence information of *B. microti* gene fragments and primers of in-fusion clone.** | | | | | | |  |
| --- | --- | --- | --- | --- | --- | --- | --- |
| **Codes** | **GenBank** | **Gene length(bp)** | **Primer names** | **Nucleic acid sequences(5'-3')** | **Remarks** | |  |
| Bm 2D-1 | CCF76139 | 267 | Bm 2D-1 F | *GGGCGGATATCTCGAG*ATGGACCTCACCCTTTTA | 2D 30days NO introns | |  |
|  |  |  | Bm 2D-1 R | *GCGGTACCCGGGATCCTTA*TCATGTCATAGTATAGTTATTAAAAG | | |  |
| Bm 2D-2 | CCF74681 | 1131 | Bm 2D-2 F | *GGGCGGATATCTCGAG*ATGGGTGATGATGACAATG | 2D 30days NO introns | |  |
|  |  |  | Bm 2D-2 R | *GCGGTACCCGGGATCCTTA*CTAGAAGCACTTTCGGT |  | |  |
| Bm 2D-3 | CCF72738 | 333 | Bm 2D-3 F | *GGGCGGATATCTCGAG*AAATATATTCCGCTGGCG | 2D 30days NO introns | |  |
|  |  |  | Bm 2D-3 R | *GCGGTACCCGGGATCCTTA*TCAGGGTAATAGCTTAGC |  | |  |
| Bm 2D-4 | CCF72738 | 690 | Bm 2D-4 F | *GGGCGGATATCTCGAG*ATGTTGGAAACTTCAAGTG | 2D 30days NO introns | |  |
|  |  |  | Bm 2D-4 R | *GCGGTACCCGGGATCCTTA*CTTGTATAAATCCACCAAAGT |  | |  |
| Bm 2D-5 | AAO18095 | 759 | Bm 2D-5 F | *GGGCGGATATCTCGAG*TATGCAAAATTTTCTGAACAGAT | | 2D 30days NO introns | |
|  |  |  | Bm 2D-5 R | *GCGGTACCCGGGATCCTTA*TTAATTTAGAATTAATATTAATGCAGT | | |  |
| Bm 2D-6 | BAH28858 | 849 | Bm 2D-6 F | *GGGCGGATATCTCGAG*GTGTTTTTATTCTTGGGATTTTT | 2D 30days NO introns | |  |
|  |  |  | Bm 2D-6 R | *GCGGTACCCGGGATCCTTA*ATTGGTCAACCTCCCTA |  | |  |
| Bm 2D-7 | BAH28858 | 1062 | Bm 2D-7 F | *GGGCGGATATCTCGAG*GTTATCATCTGCCACAG | 2D 30days NO introns | |  |
|  |  |  | Bm 2D-7 R | *GCGGTACCCGGGATCCTTA*TCATAACTCATCGCCATAA |  | |  |
| Bm 2D-8 | CCF72475 | 306 | Bm 2D-8 F | *GGGCGGATATCTCGAG*ATGATACTTAGTGAAAATCCGT | 2D 30days NO introns | |  |
|  |  |  | Bm 2D-8 R | *GCGGTACCCGGGATCCTTA*AGATTTGAAGCTTGGAATAGT |  | |  |
| Bm 2D-9 | CCF72520 | 933 | Bm 2D-9 F | *GGGCGGATATCTCGAG*GATGTTGTTGCAATAAATGAC | 2D 30days NO introns | |  |
|  |  |  | Bm 2D-9 R | *GCGGTACCCGGGATCCTTA*CTATTGTTTAGATTGGACAAAAG |  | |  |
| Bm 2D-10 | CCF72691 | 1158 | Bm 2D-10 F | *GGGCGGATATCTCGAG*ATGCACTCGAATATAGTTAC | 2D 30days NO introns | |  |
|  |  |  | Bm 2D-10 R | *GCGGTACCCGGGATCCTTA*GGCCCTTAAAGCCTTG |  | |  |
| Bm 2D-11 | CCF72691 | 1389 | Bm 2D-11 F | *GGGCGGATATCTCGAG*GATGTTGAAGGCTACCA | 2D 30days NO introns | |  |
|  |  |  | Bm 2D-11 R | *GCGGTACCCGGGATCCTTA*GTAGTAACTGGCTAGC |  | |  |
| Bm 2D-12 | CCF73033 | 1536 | Bm 2D-12 F | *GGGCGGATATCTCGAG*ATGTATGACGCTGAAAACAT | 2D 30days NO introns | |  |
|  |  |  | Bm 2D-12 R | *GCGGTACCCGGGATCCTTA*TTACAGCTTAACGGTTATTATAT |  | |  |
| Bm 2D-13 | CCF73151 | 456 | Bm 2D-13 F | *GGGCGGATATCTCGAG*ATGCCCGTGACGTTC | 2D 30days NO introns | |  |
|  |  |  | Bm 2D-13 R | *GCGGTACCCGGGATCCTTA*TGTACTGGGGAAGTGTA |  | |  |
| Bm 2D-14 | BAF02622 | 1251 | Bm 2D-14 F | *GGGCGGATATCTCGAG*ATGAAGTCGTTATCTTTAATACG | 2D 30days NO introns | |  |
|  |  |  | Bm 2D-14 R | *GCGGTACCCGGGATCCTTA*AGCCTGAATAGCCGC |  | |  |
| Bm 2D-15 | CCF73197 | 1005 | Bm 2D-15 F | *GGGCGGATATCTCGAG*AAGCTGTATACTAACAGGC | 2D 30days NO introns | |  |
|  |  |  | Bm 2D-15 R | *GCGGTACCCGGGATCCTTA*TCAACTATCTTGTTCTAGG |  | |  |
| Bm 2D-16 | CCF73248 | 309 | Bm 2D-16 F | *GGGCGGATATCTCGAG*GATACCCCTAAGCTATATT | 2D 30days NO introns | |  |
|  |  |  | Bm 2D-16 R | *GCGGTACCCGGGATCCTTA*TTACGGGAAAGTGTCGAT |  | |  |
| Bm 2D-17 | CCF73248 | 327 | Bm 2D-17 F | *GGGCGGATATCTCGAG*ATGGATACACTAATTGGTATAAA | 2D 30days NO introns | |  |
|  |  |  | Bm 2D-17 R | *GCGGTACCCGGGATCCTTA*CTGTTCAAACCCTGCTA |  | |  |
| Bm 2D-18 | CCF73397 | 807 | Bm 2D-18 F | *GGGCGGATATCTCGAG*GAAATGGGTTGCCACAT | 2D 30days NO introns | |  |
|  |  |  | Bm 2D-18 R | *GCGGTACCCGGGATCCTTA*TCAGCTTGGAGCTGG |  | |  |
| Bm 2D-19 | CCF73474 | 486 | Bm 2D-19 F | *GGGCGGATATCTCGAG*ATCCCCGCGACAATC | 2D 30days NO introns | |  |
|  |  |  | Bm 2D-19 R | *GCGGTACCCGGGATCCTTA*TCAAACTAGGTCCCC |  | |  |
| Bm 2D-20 | CCF73712 | 1179 | Bm 2D-20 F | *GGGCGGATATCTCGAG*ATGATCCGACCTGTG | 2D 30days NO introns | |  |
|  |  |  | Bm 2D-20 R | *GCGGTACCCGGGATCCTTA*TTATTCGGCAATTTCTTGCT |  | |  |
| Bm 2D-21 | CCF73790 | 321 | Bm 2D-21 F | *GGGCGGATATCTCGAG*TCTTCGGTAGAATCTTGTATA | 2D 30days NO introns | |  |
|  |  |  | Bm 2D-21 R | *GCGGTACCCGGGATCCTTA*CTGTTCTGACTTGTTCTTAAT |  | |  |
| Bm 2D-22 | CCF73790 | 717 | Bm 2D-22 F | *GGGCGGATATCTCGAG*ATTTTATGGGAGAAACTCCTT | 2D 30days NO introns | |  |
|  |  |  | Bm 2D-22 R | *GCGGTACCCGGGATCCTTA*TTCCACACTAGCGCTAA |  | |  |
| Bm 2D-23 | CCF73790 | 1638 | Bm 2D-23 F | *GGGCGGATATCTCGAG*GACCCTTCATTGTGTAATTT | 2D 30days NO introns | |  |
|  |  |  | Bm 2D-23 R | *GCGGTACCCGGGATCCTTA*CTACTTCTTTCTTTTTTTGACTT |  | |  |
| Bm 2D-24 | CCF73803 | 1203 | Bm 2D-24 F | *GGGCGGATATCTCGAG*ATGGATGAATTTCAAATACCC | 2D 30days NO introns | |  |
|  |  |  | Bm 2D-24 R | *GCGGTACCCGGGATCCTTA*TTAGGTGTGATTAATCCTC |  | |  |
| Bm 2D-25 | CCF73838 | 705 | Bm 2D-25 F | *GGGCGGATATCTCGAG*GATGAAGTGGAAAAGGC | 2D 30days NO introns | |  |
|  |  |  | Bm 2D-25 R | *GCGGTACCCGGGATCCTTA*TAATAATGTTAAGGCGCGC |  | |  |
| Bm 2D-26 | CCF73865 | 255 | Bm 2D-26 F | *GGGCGGATATCTCGAG*ATATACGTATCTCTGGATC | 2D 30days NO introns | |  |
|  |  |  | Bm 2D-26 R | *GCGGTACCCGGGATCCTTA*CGTCACCTGCGTGTT |  | |  |
| Bm 2D-27 | CCF73865 | 453 | Bm 2D-27 F | *GGGCGGATATCTCGAG*GGGGACCCGATCGAC | 2D 30days NO introns | |  |
|  |  |  | Bm 2D-27 R | *GCGGTACCCGGGATCCTTA*TTAGCCACAAAGGGAAC |  | |  |
| Bm 2D-28 | CCF73865 | 525 | Bm 2D-28 F | *GGGCGGATATCTCGAG*ATTTCTTATACAGTGCTAAAGTG | 2D 30days NO introns | |  |
|  |  |  | Bm 2D-28 R | *GCGGTACCCGGGATCCTTA*AGTGTCACGGGTTATATG |  | |  |
| Bm 2D-29 | CCF74204 | 717 | Bm 2D-29 F | *GGGCGGATATCTCGAG*TATGCAAAATTTTCTGAACAGAT | | 2D 30days NO introns | |
|  |  |  | Bm 2D-29 R | *GCGGTACCCGGGATCCTTA*TTGTCCAGGGATTATTGC |  | |  |
| Bm 2D-30 | CCF74238 | 678 | Bm 2D-30 F | *GGGCGGATATCTCGAG*ATAATTTCCGATGCTTTCAG | 2D 30days NO introns | |  |
|  |  |  | Bm 2D-30 R | *GCGGTACCCGGGATCCTTA*CCTTGGGAAGTCCATTA |  | |  |
| Bm 2D-31 | LN871598 | 453 | Bm 2D-31 F | *GGGCGGATATCTCGAG*ATGTCTGACCACAGTC | 2D 30days NO introns | |  |
|  |  |  | Bm 2D-31 R | *GCGGTACCCGGGATCCTTA*GGATCTTCTGCCTCTA |  | |  |
| Bm 2D-32 | CCF74546 | 534 | Bm 2D-32 F | *GGGCGGATATCTCGAG*ATGGACTGTGAATTGTCG | 2D 30days NO introns | |  |
|  |  |  | Bm 2D-32 R | *GCGGTACCCGGGATCCTTA*CTCCATTGCTTTAGATCTTT |  | |  |
| Bm 2D-33 | CCF74637 | 432 | Bm 2D-33 F | *GGGCGGATATCTCGAG*GGATTAGAAGATGCTGTAG | 2D 30days NO introns | |  |
|  |  |  | Bm 2D-33 R | *GCGGTACCCGGGATCCTTA*GTATAGTTCCCTCTGCT |  | |  |
| Bm 2D-34 | CCF74637 | 540 | Bm 2D-34 F | *GGGCGGATATCTCGAG*AATCCCAACTTCAACGTAC | 2D 30days NO introns | |  |
|  |  |  | Bm 2D-34 R | *GCGGTACCCGGGATCCTTA*CTCTATATTCTTAACCTCCA |  | |  |
| Bm 2D-35 | CCF74637 | 678 | Bm 2D-35 F | *GGGCGGATATCTCGAG*ATTGAAAGGGAGAAACAGC | 2D 30days NO introns | |  |
|  |  |  | Bm 2D-35 R | *GCGGTACCCGGGATCCTTA*CATTTTAGAGTGAACTTCCA |  | |  |
| Bm 2D-36 | CCF74836 | 519 | Bm 2D-36 F | *GGGCGGATATCTCGAG*ATGGGTGGAGCTGTC | 2D 30days NO introns | |  |
|  |  |  | Bm 2D-36 R | *GCGGTACCCGGGATCCTTA*TTAAATTAAACCAAGTTTACTTTTCAC | | |  |
| Bm 2D-37 | CCF74956 | 1389 | Bm 2D-37 F | *GGGCGGATATCTCGAG*ATGTCGCTTTACAGCG | 2D 30days NO introns | |  |
|  |  |  | Bm 2D-37 R | *GCGGTACCCGGGATCCTTA*TCAATTAGGATGACAAGCTT |  | |  |
| Bm 2D-38 | CCF75049 | 246 | Bm 2D-38 F | *GGGCGGATATCTCGAG*GGCTCCGCTTGCACA | 2D 30days NO introns | |  |
|  |  |  | Bm 2D-38 R | *GCGGTACCCGGGATCCTTA*CTAGGTCCACCTCAT |  | |  |
| Bm 2D-39 | CCF75049 | 924 | Bm 2D-39 F | *GGGCGGATATCTCGAG*ATGGACCCTAGGGTTTT | 2D 30days NO introns | |  |
|  |  |  | Bm 2D-39 R | *GCGGTACCCGGGATCCTTA*AGAACTAACAGCAAATCCAT |  | |  |
| Bm 2D-40 | CCF75408 | 615 | Bm 2D-40 F | *GGGCGGATATCTCGAG*ATTTTGGTCTATGATCTCG | 2D 30days NO introns | |  |
|  |  |  | Bm 2D-40 R | *GCGGTACCCGGGATCCTTA*ACCCAGTGATAGCGG |  | |  |
| Bm 2D-41 | CCF75408 | 687 | Bm 2D-41 F | *GGGCGGATATCTCGAG*GGTATCGAAACTGTTGGT | | 2D 30days NO introns | |
|  |  |  | Bm 2D-41 R | *GCGGTACCCGGGATCCTTA*TCATAACTCATCCCCGT |  | |  |
| Bm 2D-42 | CCF75517 | 339 | Bm 2D-42 F | *GGGCGGATATCTCGAG*GATTTAGATATTATTTTTGACAACAG | 2D 30days NO introns | |  |
|  |  |  | Bm 2D-42 R | *GCGGTACCCGGGATCCTTA*CTCAAAGCCCACAGAAT |  | |  |
| Bm 2D-43 | CCF75640 | 192 | Bm 2D-43 F | *GGGCGGATATCTCGAG*ATGCTTGCTATTTTTCTGG | 2D 30days NO introns | |  |
|  |  |  | Bm 2D-43 R | *GCGGTACCCGGGATCCTTA*TCATGATGTGGCCGC |  | |  |
| Bm 2D-44 | CCF75648 | 1710 | Bm 2D-44 F | *GGGCGGATATCTCGAG*ATGGACTTGAACAGTGC | 2D 30days NO introns | |  |
|  |  |  | Bm 2D-44 R | *GCGGTACCCGGGATCCTTA*TTAGGTATTATACCCCCG |  | |  |
| Bm 2D-45 | CCF75859 | 165 | Bm 2D-45 F | *GGGCGGATATCTCGAG*TGGAAATACATTAAGGACCA | 2D 30days NO introns | |  |
|  |  |  | Bm 2D-45 R | *GCGGTACCCGGGATCCTTA*TTATTCCACAGGCTTTCG |  | |  |
| Bm 2D-46 | CCF75859 | 244 | Bm 2D-46 F | *GGGCGGATATCTCGAG*ATGACAGTGACACTTTTTTC | 2D 30days NO introns | |  |
|  |  |  | Bm 2D-46 R | *GCGGTACCCGGGATCCTTA*ACACAAGTTTGACTGCC |  | |  |
| Bm 2D-47 | CCF75913 | 777 | Bm 2D-47 F | *GGGCGGATATCTCGAG*ATGGATGGATTTAAGGATAC | 2D 30days NO introns | |  |
|  |  |  | Bm 2D-47 R | *GCGGTACCCGGGATCCTTA*TCATTTCAGATGGATATAAGG |  | |  |
| Bm 2D-48 | CCF75944 | 195 | Bm 2D-48 F | *GGGCGGATATCTCGAG*ATGTTTGGCGATGAAGG | 2D 30days NO introns | |  |
|  |  |  | Bm 2D-48 R | *GCGGTACCCGGGATCCTTA*TTAATTGCTGGCGGCC |  | |  |
| Bm 2D-49 | CCF75956 | 255 | Bm 2D-49 F | *GGGCGGATATCTCGAG*ATGGGCTTGGTGATTG | 2D 30days NO introns | |  |
|  |  |  | Bm 2D-49 R | *GCGGTACCCGGGATCCTTA*TTATTGCGTACTCTTCCATTT |  | |  |
| Bm 2D-50 | BAF02621 | 573 | Bm 2D-50 F | *GGGCGGATATCTCGAG*ATGTCTGGGCCAGCT | 2D 30days NO introns | |  |
|  |  |  | Bm 2D-50 R | *GCGGTACCCGGGATCCTTA*AGAAGTACCTTTCTTGTCC |  | |  |
| Bm 2D-51 | BAF02621 | 1350 | Bm 2D-51 F | *GGGCGGATATCTCGAG*GTGTTGTTTTTCGATTTGG | 2D 30days NO introns | |  |
|  |  |  | Bm 2D-51 R | *GCGGTACCCGGGATCCTTA*TTAATCAACTTCCTCGACA |  | |  |
| Bm 2D-52 | CCF76134 | 1539 | Bm 2D-52 F | *GGGCGGATATCTCGAG*ATGTCTAATGATGGAAAGAAAG | 2D 30days NO introns | |  |
|  |  |  | Bm 2D-52 R | *GCGGTACCCGGGATCCTTA*TCAAGCTTCTTTGACCTG |  | |  |
| Bm 2D-53 | BAF02622 | 792 | Bm 2D-53 F | *GGGCGGATATCTCGAG*GGCGTGCTAAAGGGC | 2D 30days NO introns | |  |
|  |  |  | Bm 2D-53 R | *GCGGTACCCGGGATCCTTA*TTAGTCAAATGCAATTTCATAAAGA | | |  |
| Bm 2D-54 | CCF72650 | 318 | Bm 2D-54 F | *GGGCGGATATCTCGAG*GATCCTTTCAACCCTCT | 2D 7days NO introns | |  |
|  |  |  | Bm 2D-54 R | *GCGGTACCCGGGATCCTTA*GCTGGAATAAGTCGG |  | |  |
| Bm 2D-55 | CCF74285 | 291 | Bm 2D-55 F | *GGGCGGATATCTCGAG*GAAATGGCTGAGGCTAT | 2D 7days NO introns | |  |
|  |  |  | Bm 2D-55 R | *GCGGTACCCGGGATCCTTA*CGTAGTATTAGGAATTAGCTT |  | |  |
| Bm 2D-56 | CCF74336 | 327 | Bm 2D-56 F | *GGGCGGATATCTCGAG*AAGTTGAAATACAAGGGCAAT | 2D 7days NO introns | |  |
|  |  |  | Bm 2D-56 R | *GCGGTACCCGGGATCCTTA*TTTTGGTTTATCTCCATCAATTTTA |  | |  |
| Bm 2D-57 | CCF74416 | 306 | Bm 2D-57 F | *GGGCGGATATCTCGAG*ATGCATTGTAGGCAATTGC | 2D 7days NO introns | |  |
|  |  |  | Bm 2D-57 R | *GCGGTACCCGGGATCCTTA*TTTGGGCGGGAATTCTT |  | |  |
| Bm 2D-58 | CCF74416 | 486 | Bm 2D-58 F | *GGGCGGATATCTCGAG*ATCGCATCTTACGCTGA | 2D 7days NO introns | |  |
|  |  |  | Bm 2D-58 R | *GCGGTACCCGGGATCCTTA*TTACCTATGGAGATTGATCTT |  | |  |
| Bm 2D-59 | CCF75986 | 2118 | Bm 2D-59 F | *GGGCGGATATCTCGAG*TCATGTAAAACTGGCATGG | 2D 7days NO introns | |  |
|  |  |  | Bm 2D-59 R | *GCGGTACCCGGGATCCTTA*GCCCTTTATATATTTAACCG |  | |  |
| Bm 2D-60 | CCF73794.1 | 198 | Bm 2D-60 F | *GGGCGGATATCTCGAG*ACTAGCTGCTCATTGTTT | 2D 30days NO introns | |  |
|  |  |  | Bm 2D-60 R | *GCGGTACCCGGGATCCTTA*AATTTTGAGAGTAGCA |  | |  |
| Bm 2D-61 | CCF73794.1 | 174 | Bm 2D-61 F | *GGGCGGATATCTCGAG* ATGTACTATGTGGATTGTAAAAATACTG | 2D 30days NO introns | |  |
|  |  |  | Bm 2D-61 R | *GCGGTACCCGGGATCCTTA* AGTTTGAGGCACTA |  | |  |
| Bm 2D-62 | CCF73388.1 | 201 | Bm 2D-62 F | *GGGCGGATATCTCGAG* TGTCAACGTAAATGTCATGA | 2D 30days NO introns | |  |
|  |  |  | Bm 2D-62 R | *GCGGTACCCGGGATCCTTA* GTTATTCGTCATGTTAG |  | |  |
| Bm 2D-63 | CCF72700.1 | 570 | Bm 2D-63 F | *GGGCGGATATCTCGAG* GCAAACGATTGGTCA | 2D 30days NO introns | |  |
|  |  |  | Bm 2D-63 R | *GCGGTACCCGGGATCCTTA* AACCTTTTGAATGC |  | |  |
| Bm 2D-64 | CCF72700.1 | 252 | Bm 2D-64 F | *GGGCGGATATCTCGAG* GATACAAAAAGCGCTG | 2D 30days NO introns | |  |
|  |  |  | Bm 2D-64 R | *GCGGTACCCGGGATCCTTA* CACTAATCGCTTTAG |  | |  |
| Bm 2D-65 | CCF72700.1 | 453 | Bm 2D-65 F | *GGGCGGATATCTCGAG* ATGAGGGCGACAATTAA | 2D 30days NO introns | |  |
|  |  |  | Bm 2D-65 R | *GCGGTACCCGGGATCCTTA* CACTAATCGCTTTAG |  | |  |
| Bm 2D-66 | CCF72700.1 | 570 | Bm 2D-66 F | *GGGCGGATATCTCGAG* GTTATCGACTATGGGCAGAAGAAA | 2D 30days NO introns | |  |
|  |  |  | Bm 2D-66 R | *GCGGTACCCGGGATCCTTA* GCACCTCCCCAATTT |  | |  |
| Bm 2D-67 | CCF73920.1 | 297 | Bm 2D-67 F | *GGGCGGATATCTCGAG* GTCAATAAAACGGAAAAGGT | 2D 30days NO introns | |  |
|  |  |  | Bm 2D-67 R | *GCGGTACCCGGGATCCTTA* TAAATATGATGTACTCGTACC |  | |  |
| Bm 2D-68 | CCF73920.1 | 348 | Bm 2D-68 F | *GGGCGGATATCTCGAG* GAAACTAGCATTGGGC | 2D 30days NO introns | |  |
|  |  |  | Bm 2D-68 R | *GCGGTACCCGGGATCCTTA* CTTGAATTCGTTCAATAATT |  | |  |
| Bm 2D-69 | CCF72705.1 | 702 | Bm 2D-69 F | *GGGCGGATATCTCGAG*  AAGGATTACCATGCCAC | 2D 30days NO introns | |  |
|  |  |  | Bm 2D-69 R | *GCGGTACCCGGGATCCTTA* TGCAGGTGAATCTTC |  | |  |
| Bm 2D-70 | CCF72705.1 | 177 | Bm 2D-70 F | *GGGCGGATATCTCGAG* AAGATAACCGATCAGGATAG G | 2D 30days NO introns | |  |
|  |  |  | Bm 2D-70 R | *GCGGTACCCGGGATCCTTA* TATATGGTGTTCCGATT |  | |  |
| Bm 2D-71 | CCF72705.1 | 573 | Bm 2D-71 F | *GGGCGGATATCTCGAG* GATATACACACTTTGCTTGTG | 2D 30days NO introns | |  |
|  |  |  | Bm 2D-71 R | *GCGGTACCCGGGATCCTTA* TCCTTCACTGGGCT |  | |  |
| Bm 2D-72 | CCF74749.1 | 414 | Bm 2D-72 F | *GGGCGGATATCTCGAG* TTTGTTAATCTCAACACGATGG | 2D 30days NO introns | |  |
|  |  |  | Bm 2D-72 R | *GCGGTACCCGGGATCCTTA* TTCACCTTTCCACAA |  | |  |
| Bm 2D-73 | CCF74749.1 | 546 | Bm 2D-73 F | *GGGCGGATATCTCGAG* CAGAGATCAGCATACATTTC | 2D 30days NO introns | |  |
|  |  |  | Bm 2D-73 R | *GCGGTACCCGGGATCCTTA* GGAGTTGAAGAGAGA |  | |  |
| Bm 2D-74 | CCF75374.1 | 606 | Bm 2D-74 F | *GGGCGGATATCTCGAG* ATGTTTGTGGTAAACCGGAA | 2D 30days NO introns | |  |
|  |  |  | Bm 2D-74R | *GCGGTACCCGGGATCCTTA* TGTGGGAGTGGCG |  | |  |
| Bm 2D-75 | CCF75374.1 | 1191 | Bm 2D-75 F | *GGGCGGATATCTCGAG* CATTCCAATGGGATTATACCTA | 2D 30days NO introns | |  |
|  |  |  | Bm 2D-75R | *GCGGTACCCGGGATCCTTA* GTCATTCCAGAGATC |  | |  |
| Bm 2D-76 | CCF73895.1 | 918 | Bm 2D-76 F | *GGGCGGATATCTCGAG* AAAAGTCCCAGCCCAATTAA | 2D 30days NO introns | |  |
|  |  |  | Bm 2D-76R | *GCGGTACCCGGGATCCTTA* TTGTCCAGACCTAACT |  | |  |
| Bm 2D-77 | CCF73895.1 | 369 | Bm 2D-77 F | *GGGCGGATATCTCGAG* GGCTGCGTTATTTGC | 2D 30days NO introns | |  |
|  |  |  | Bm 2D-77R | *GCGGTACCCGGGATCCTTA* ATCAGTAATGATCTTAGTAATTT |  | |  |
| Bm 2D-78 | CCF75101.1 | 750 | Bm 2D-78 F | *GGGCGGATATCTCGAG* ATGTCAAGCAGTGAAAGTAATAC | 2D 30days NO introns | |  |
|  |  |  | Bm 2D-78R | *GCGGTACCCGGGATCCTTA* ATACCTGTCCAGCGT |  | |  |
| Bm 2D-79 | CCF75101.1 | 822 | Bm 2D-79 F | *GGGCGGATATCTCGAG* TCTCTCATTAATTGCCCTATTGC | 2D 30days NO introns | |  |
|  |  |  | Bm 2D-79R | *GCGGTACCCGGGATCCTTA* GAGAGGAAAAGGGC |  | |  |
| Bm 2D-80 | CCF75101.1 | 879 | Bm 2D-80 F | *GGGCGGATATCTCGAG* AACCCAGATATTTCAATTCAGGG | 2D 30days NO introns | |  |
|  |  |  | Bm 2D-80R | *GCGGTACCCGGGATCCTTA* TTTTAAATAGAGTGCAAGCC |  | |  |
| Bm 2D-81 | CCF75298.1 | 858 | Bm 2D-81 F | *GGGCGGATATCTCGAG* ATGGAAAATAGCAATACATATAAACTT | 2D 30days NO introns | |  |
|  |  |  | Bm 2D-81R | *GCGGTACCCGGGATCCTTA* ATTTGATTTATCATACAAGT |  | |  |
| Bm 2D-82 | CCF75298.1 | 1080 | Bm 2D-82 F | *GGGCGGATATCTCGAG* CCATTTGATTTATCATACAAGGATA | 2D 30days NO introns | |  |
|  |  |  | Bm 2D-82R | *GCGGTACCCGGGATCCTTA* TATTTTTGTGAGTTTCCT |  | |  |
| Bm 2D-83 | CCF75422.1 | 327 | Bm 2D-83 F | *GGGCGGATATCTCGAG* CTCCTTAGCATATGGG | 2D 30days NO introns | |  |
|  |  |  | Bm 2D-83R | *GCGGTACCCGGGATCCTTA* GTTTACAATTGTAAATGC |  | |  |
| Bm 2D-84 | CCF73602.1 | 609 | Bm 2D-84 F | *GGGCGGATATCTCGAG* AAAAAAGAGTTATTGGACGAAATT | 2D 30days NO introns | |  |
|  |  |  | Bm 2D-84R | *GCGGTACCCGGGATCCTTA* TTTGCGGAGAAGG |  | |  |
| Bm 2D-85 | CCF73722.1 | 267 | Bm 2D-85 F | *GGGCGGATATCTCGAG* TCAAAGGATGAAAATGGCAA | 2D 30days NO introns | |  |
|  |  |  | Bm 2D-85R | *GCGGTACCCGGGATCCTTA* ACTATTTTTAGTATCATGCTG |  | |  |
| Bm 2D-86 | CCF73722.1 | 303 | Bm 2D-86F | *GGGCGGATATCTCGAG* TATTTCAAAGATGAATTTGACCAAGC | 2D 30days NO introns | |  |
|  |  |  | Bm 2D-86R | *GCGGTACCCGGGATCCTTA* ATTTGCTCGTTTGG |  | |  |
| Bm 2D-87 | CCF73331.1 | 309 | Bm 2D-87F | *GGGCGGATATCTCGAG* ATGGCTGCTTACAATTCAAT | 2D 30days NO introns | |  |
|  |  |  | Bm 2D-87R | *GCGGTACCCGGGATCCTTA* GAGACAACCATTAAGAT |  | |  |
| Bm 2D-88 | CCF73488.1 | 369 | Bm 2D-88F | *GGGCGGATATCTCGAG* GTTGCCGCTTTATGG | 2D 30days NO introns | |  |
|  |  |  | Bm 2D-88R | *GCGGTACCCGGGATCCTTA* CCGATACAAGATGC |  | |  |
| Bm 2D-89 | CCF74961.1 | 489 | Bm 2D-89F | *GGGCGGATATCTCGAG* AGGAAGAAACAGAGGGAAAA | 2D 30days NO introns | |  |
|  |  |  | Bm 2D-89R | *GCGGTACCCGGGATCCTTA* CAAGTTTAGGTTGGTA |  | |  |
| Bm 2D-90 | CCF74961.1 | 1134 | Bm 2D-90F | *GGGCGGATATCTCGAG* CTAAAAAGAGCAACTGAATGT | 2D 30days NO introns | |  |
|  |  |  | Bm 2D-90R | *GCGGTACCCGGGATCCTTA* CTCCACTTTCACG |  | |  |
| Bm 2D-91 | CCF74961.1 | 306 | Bm 2D-91F | *GGGCGGATATCTCGAG* ACCTCGGGTTATTTATGGG | 2D 30days NO introns | |  |
|  |  |  | Bm 2D-91R | *GCGGTACCCGGGATCCTTA* ATATCCTCGTGTTGG |  | |  |
| Bm 2D-92 | CCF74546.1 | 534 | Bm 2D-92F | *GGGCGGATATCTCGAG* ATGGACTGTGAATTGTCG | 2D 30days NO introns | |  |
|  |  |  | Bm 2D-92R | *GCGGTACCCGGGATCCTTA* CTCCATTGCTTTAGAT |  | |  |
| Bm 2D-93 | CCF75011.1 | 246 | Bm 2D-93F | *GGGCGGATATCTCGAG*AAAACAATATTTTCAGAAATAGTTAATCTC | 2D 30days NO introns | |  |
|  |  |  | Bm 2D-93R | *GCGGTACCCGGGATCCTTA*  AAAAGGAATTTTAGCC |  | |  |
| Bm 2D-94 | CCF75011.1 | 246 | Bm 2D-94F | *GGGCGGATATCTCGAG* GAAATGAAGCAAATTGCTAC | 2D 30days NO introns | |  |
|  |  |  | Bm 2D-94R | *GCGGTACCCGGGATCCTTA* ATCAAAGGGTTCGAAAT |  | |  |
| Bm 2D-95 | CCF75281.1 | 918 | Bm 2D-95F | *GGGCGGATATCTCGAG* ATGGCAACAAACGATACTTA | 2D 30days NO introns | |  |
|  |  |  | Bm 2D-95R | *GCGGTACCCGGGATCCTTA* CTGAACAAGTTTTGATT |  | |  |
| Bm 2D-96 | CCF75281.1 | 849 | Bm 2D-96F | *GGGCGGATATCTCGAG* GAATACCAACAGTCTTTGG | 2D 30days NO introns | |  |
|  |  |  | Bm 2D-96R | *GCGGTACCCGGGATCCTTA* AATATTACACTTTCCATGC |  | |  |
| Bm 2D-97 | CCF75281.1 | 984 | Bm 2D-97F | *GGGCGGATATCTCGAG* CACTTATATTCGCTAAAACG | 2D 30days NO introns | |  |
|  |  |  | Bm 2D-97R | *GCGGTACCCGGGATCCTTA*  TGGATCTTTAGAAGTG |  | |  |
| Bm 2D-98 | CCF72679.1 | 330 | Bm 2D-98F | *GGGCGGATATCTCGAG* ATGTGGTGTGCCAC | 2D 30days NO introns | |  |
|  |  |  | Bm 2D-98R | *GCGGTACCCGGGATCCTTA* ATCTGATATCGGACC |  | |  |
| Bm 2D-99 | CCF72679.1 | 375 | Bm 2D-99F | *GGGCGGATATCTCGAG* TTAATCACTGGAGCTGC | 2D 30days NO introns | |  |
|  |  |  | Bm 2D-99R | *GCGGTACCCGGGATCCTTA* CTTGCCAAGCAC |  | |  |
| Bm 2D-100 | CCF75929.1 | 237 | Bm 2D-100 | ATGATAGTACCGATCCGATGTTTCACTTGTGGAAAGGTTATTGGTAATTTATGGAATAAATGGTTGGATAAATTATCTAGAGATATTCCAGAAGGGCAGGCTCTGGATGAATTGGGCCTATCTAGATATTGTTGCCGTAGGATGATACTAACCCATGTTGATTTGATTGATAAATTACTGGCATATAATAGTAAGTTAATTAATAATGAAGTTTACGAAAAGAGGACTATTAATAGT | 2D 30days NO introns  Synthesis | |  |
| Bm 2D-101 | CCF76062.1 | 309 | Bm 2D-101F | F: *GGGCGGATATCTCGAG* TGAGAGAAATAATAAGTATACACGT | 2D 30days NO introns | |  |
|  |  |  | Bm 2D-101R | R: *GCGGTACCCGGGATCCTTA* AAAGTTGTTTGCGG |  | |  |
| Bm 2D-102 | CCF76062.1 | 636 | Bm 2D-102F | F: *GGGCGGATATCTCGAG* CGGCCCAATTATACTAAT | 2D 30days NO introns | |  |
|  |  |  | Bm 2D-102R | R: *GCGGTACCCGGGATCCTTA* GTAATCATTTTCTAGCGT |  | |  |
| Bm 2D-103 | CCF73594.1 | 189 | Bm 2D-103 | ATGAGTGTGGAAGATGTGGAAGATGTGGAATGTGGCATAAC  AAACTCAATTGTGTTTATCATGCACTGCACAAAAATATCAAATAATATAGTCAATTACATTCGAATGATGCACATTGCTGTGTGCACATGTGTGCTCACCACTTCTAAATACACGGACGTGCCAACTAAAACAACAACAATACAAATA | 2D 30days NO introns Synthesis | |  |
| Bm 2D-104 | BAH22737.1 | 708 | Bm 2D-104F | *GGGCGGATATCTCGAG* ATGGTAGTGGATGCTGTTAA | 2D 30days NO introns | |  |
|  |  |  | Bm 2D-104R | *GCGGTACCCGGGATCCTTA* TTCAATGGCCCCT |  | |  |
| Bm 2D-105 | BAB83929.1 | 1482 | Bm 2D-105F | *GGGCGGATATCTCGAG* GTGACAAATTGCATTAAAACG | 2D 30days NO introns | |  |
|  |  |  | Bm 2D-105R | *GCGGTACCCGGGATCCTTA* TTGCTGCTTTATCGT |  | |  |
| Bm 2D-106 | CCF72846.1 | 720 | Bm 2D-106F | *GGGCGGATATCTCGAG*  ATGATCAAAACATCTGAGGTGG | 2D 30days NO introns | |  |
|  |  |  | Bm 2D-106R | *GCGGTACCCGGGATCCTTA* CCATAGATATAATGCTTGTT |  | |  |
| Bm 2D-107 | CCF72846.1 | 993 | Bm 2D-107F | *GGGCGGATATCTCGAG* AAATATGTATCTGAAAAATTGGCTG | 2D 30days NO introns | |  |
|  |  |  | Bm 2D-107R | *GCGGTACCCGGGATCCTTA* ATTGTGTGTAAAATTATAGTAAATC | | |  |
| Bm 2D-108 | CCF72846.1 | 723 | Bm 2D-108F | *GGGCGGATATCTCGAG* CTAGATGATCGATTACTTAGCTTCA | 2D 30days NO introns | |  |
|  |  |  | Bm 2D-108R | *GCGGTACCCGGGATCCTTA* AGTAAAGCTCACCCTT |  | |  |
| Bm 2D-109 | CCF72898.1 | 1494 | Bm 2D-109F | *GGGCGGATATCTCGAG* ATGAACCAACAAATGAGTGTGT | 2D 30days NO introns | |  |
|  |  |  | Bm 2D-109R | *GCGGTACCCGGGATCCTTA* GATTTCAAGTTCTTTCTC |  | |  |
| Bm 2D-110 | CCF72898.1 | 465 | Bm 2D-110F | *GGGCGGATATCTCGAG* ATAACTGTTATGGGTAACAGCG | 2D 30days NO introns | |  |
|  |  |  | Bm 2D-110R | *GCGGTACCCGGGATCCTTA* CGACCTTTTTGCATCATTAATAAA |  | |  |
| Bm 2D-111 | CCF75326.1 | 360 | Bm 2D-111F | *GGGCGGATATCTCGAG* GTATTATACAATGTAGATAGTTGTGGC | 2D 30days NO introns | |  |
|  |  |  | Bm 2D-111R | *GCGGTACCCGGGATCCTTA* GACAATTTGTACTTTGTCGTT |  | |  |
| Bm 2D-112 | CCF74240.1 | 1266 | Bm 2D-112F | *GGGCGGATATCTCGAG* ATGGAACATAGTGTGGTTTC | 2D 30days NO introns | |  |
|  |  |  | Bm 2D-112R | *GCGGTACCCGGGATCCTTA* CTCTATAAAATTGCACAG |  | |  |
| Bm 2D-113 | CCF72674.1 | 270 | Bm 2D-113F | *GGGCGGATATCTCGAG* ATGTCAGCCAACCAG | 2D 30days NO introns | |  |
|  |  |  | Bm 2D-113R | *GCGGTACCCGGGATCCTTA* AACGGAAATTATTTCGTG |  | |  |
| Bm 2D-114 | CCF72674.1 | 333 | Bm 2D-114F | *GGGCGGATATCTCGAG* GACCCTGCGTTTTATCC | 2D 30days NO introns | |  |
|  |  |  | Bm 2D-114R | *GCGGTACCCGGGATCCTTA* CTGTCGAGCATCTGA |  | |  |
| Bm 2D-115 | CCF73504.1 | 168 | Bm 2D-115F | *GGGCGGATATCTCGAG* ATGCCTACCGGAGCT | 2D 30days NO introns | |  |
|  |  |  | Bm 2D-115R | *GCGGTACCCGGGATCCTTA* CTTTTTTTTGGCATTTTTCTTTG |  | |  |
| Bm 2D-116 | CCF76124.1 | 258 | Bm 2D-116F | *GGGCGGATATCTCGAG* TCATTTGCACACAGGG | 2D 30days NO introns | |  |
|  |  |  | Bm 2D-116R | *GCGGTACCCGGGATCCTTA* CAATGAATTGCCCATAAAT |  | |  |
| Bm 2D-117 | CCF75386.1 | 195 | Bm 2D-117F | *GGGCGGATATCTCGAG* CTTTTTGCCAAAGCTTTGAAG | 2D 30days NO introns | |  |
|  |  |  | Bm 2D-117R | *GCGGTACCCGGGATCCTTA* GTCAAAGAGTGAAAATCC |  | |  |
| Bm 2D-118 | CCF72966.1 | 504 | Bm 2D-118F | *GGGCGGATATCTCGAG* TTCTCAAGTTCATTCTTGGATTCAA | 2D 30days NO introns | |  |
|  |  |  | Bm 2D-118R | *GCGGTACCCGGGATCCTTA* GGCTGCAATTTTAACT |  | |  |
| Bm 2D-119 | CCF72966.1 | 453 | Bm 2D-119F | *GGGCGGATATCTCGAG* CCCAGCAATGGTACCA | 2D 30days NO introns | |  |
|  |  |  | Bm 2D-119R | *GCGGTACCCGGGATCCTTA* TGTGTTGGCGTTTG |  | |  |
| Bm 2D-120 | BAF02621.1 | 1155 | Bm 2D-120F | *GGGCGGATATCTCGAG* ATGTCTGGGCCAGCTATT | 2D 30days NO introns | |  |
|  |  |  | Bm 2D-120R | *GCGGTACCCGGGATCCTTA* TTGTTCGCCAGTTAAAATT |  | |  |
| Bm 2D-121 | BAF02621.1 | 774 | Bm 2D-121F | *GGGCGGATATCTCGAG* TCTAGCAAGGTCCAAGATTTACT | 2D 30days NO introns | |  |
|  |  |  | Bm 2D-121R | *GCGGTACCCGGGATCCTTA* ATCAACTTCCTCGACA |  | |  |
| Bm 2D-122 | AAC47456.1 | 1155 | Bm 2D-122F | *GGGCGGATATCTCGAG* ATGTCTCAAG GTCCAGCTATT | 2D 30days NO introns | |  |
|  |  |  | Bm 2D-122R | *GCGGTACCCGGGATCCTTA* CTCACCACAAAGAATAG |  | |  |
| Bm 2D-123 | AAC47456.1 | 780 | Bm 2D-123F | *GGGCGGATATCTCGAG* CAATCAAGCAAGGTGCAA | 2D 30days NO introns | |  |
|  |  |  | Bm 2D-123R | *GCGGTACCCGGGATCCTTA* GTCAACTTCT TCAACAGT |  | |  |
| Bm 2D-124 | AAC47456.1 | 1251 | Bm 2D-124F | *GGGCGGATATCTCGAG* ATGAAGTCGTTATCTTTAATACGCCC | 2D 30days NO introns | |  |
|  |  |  | Bm 2D-124R | *GCGGTACCCGGGATCCTTA* AGCCTGAATAGCCGC |  | |  |
| Bm 2D-125 | AAC47456.1 | 786 | Bm 2D-125F | *GGGCGGATATCTCGAG* GTGCTAAAGG GCGAAATTAAG | 2D 30days NO introns | |  |
|  |  |  | Bm 2D-125R | *GCGGTACCCGGGATCCTTA* GTCAAATGCAATTTCATAAAGAT |  | |  |
| Bm 2D-126 | CCF74602.1 | 237 | Bm 2D-126F | *GGGCGGATATCTCGAG* ATGACTGTGTACGAACGGT | 2D 7days NO introns | |  |
|  |  |  | Bm 2D-126R | *GCGGTACCCGGGATCCTTA* TGGCATTTTT TTGTAAATGCTATA |  | |  |
| Bm 2D-127 | CCF75994.1 | 186 | Bm 2D-127F | *GGGCGGATATCTCGAG* CTTCCTTCTG GTAAAAGATTCTTTG | 2D 7days NO introns | |  |
|  |  |  | Bm 2D-127R | *GCGGTACCCGGGATCCTTA* AAGCTGTTTAAGCTGATAAA |  | |  |
| Bm 2D-128 | CCF73918.1 | 270 | Bm 2D-128F | *GGGCGGATATCTCGAG* TTTGCCTTTC GTGACAACT | 2D 7days NO introns | |  |
|  |  |  | Bm 2D-128R | *GCGGTACCCGGGATCCTTA* CTTCAACAAT CGTTG |  | |  |

The italic bases represent In-Fusion PCR primers.
