# Supplementary material for: Screening for biomarkers reflecting the progression of Babesia microti infection
Source: Parasit Vectors. 2018 Jul 3;11:379. doi: 10.1186/s13071-018-2951-0 (PMC6029176; doi:10.1186/s13071-018-2951-0)
Supplement: Supplementary file 4 — Table S4. The primer sequences of B. microti genes in prokaryotic cloning and expression. (DOCX 14 kb) [file 13071_2018_2951_MOESM4_ESM.docx]

**Additional file 4: Table S4. The primer sequences of *B. microti* genes in prokaryotic cloning and expression**

| Primer names | Nucleic acid sequences(5'-3') |
| --- | --- |
| Bm2D33F | GGGATATCGG *GGATCC* GGATTAGAAGATGCTGTAG |
| Bm2D33R | GGTGGTGGTG *CTCGAG* GTATAGTTCCCTCTGCT |
| Bm7F | AAT *GGATCC* ATGCATATCAACTACAAATTAATTAT |
| Bm7R | AAT *CTCGAG* AGCAGCATTAGGTGTGTGATT |
| Bm2D41F | TGGTGGCCAA *GGATCC* ATGACTAAGATTATCCCCCG |
| Bm2D41R | GGTGGTGGTG *CTCGAG* TGCGGCCGCAAGCTTTCAT |
| Bm2D97F | AATGGGTCGC *GGATCC* CACTTATATTCGCTAAAACG |
| Bm2D97R | GGTGGTGGTG *CTCGAG* TGGATCTTTAGAAGTG |

The italic bases represent restriction enzyme sites.
