# Supplementary material for: Screening for biomarkers reflecting the progression of Babesia microti infection
Source: Parasit Vectors. 2018 Jul 3;11:379. doi: 10.1186/s13071-018-2951-0 (PMC6029176; doi:10.1186/s13071-018-2951-0)
Supplement: Supplementary file 5 — Table S5. The primer sequences of P. vivax and P. falciparum. (DOCX 14 kb) [file 13071_2018_2951_MOESM5_ESM.docx]

**Additional file 5: Table S5. The primer sequences of *P. vivax and P. falciparum***

| Primer names | Nucleic acid sequences(5'-3') |
| --- | --- |
| rPLU1 | TCAAAGATTAAGCCATGCAAGTGA |
| rPLU5 | CCTGTTGTTGCCTTAAACTCC |
| rVIV1 | CGCTTCTAGCTTAATCCACATAACTGATAC |
| rVIV2 | ACTTCCAAGCCGAAGCAAAGAAAGTCCTTA |
| rFAL1 | ATAACATAGTTGTACGTTAAGAATAACCGC |
| rFAL2 | AAAATTCCCATGCATAAAAAATTATACAAA |
