# Supplementary figures and images for: Screening for biomarkers reflecting the progression of Babesia microti infection
Source: Parasit Vectors. 2018 Jul 3;11:379. doi: 10.1186/s13071-018-2951-0 (PMC6029176; doi:10.1186/s13071-018-2951-0)

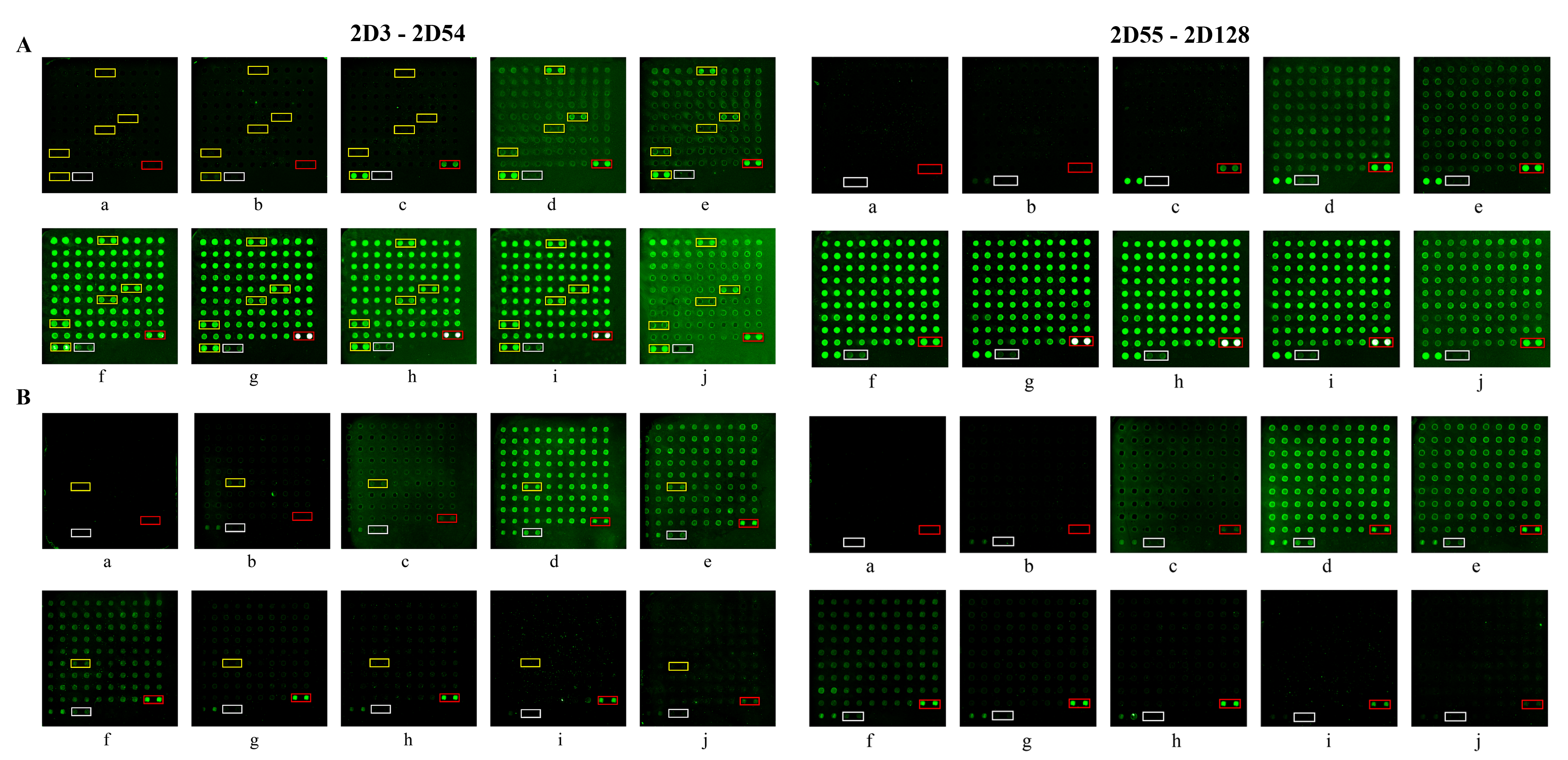

Supplement: Supplementary file 6 — Figure S1. Antibody profiling of B. microti proteins by protein arrays. Crude B. microti proteins (2D3-2D54, 2D55-2D128) react with plasma samples across ten different time points (a-j: 0, 3, 7, 14, 21, 30, 60, 120, 150 and 270 dpi). The reactions were detected with anti-mouse IgG (A) and anti-mouse IgM (B). Control reactions of wheat germ lysate that lacked vector templates (white box) and reactions of purified recombinant proteins (red box) served as negative and positive controls. Well characterized reactions with target proteins are marked with orange boxes. (TIF 3404 kb) [file 13071_2018_2951_MOESM6_ESM.tif]

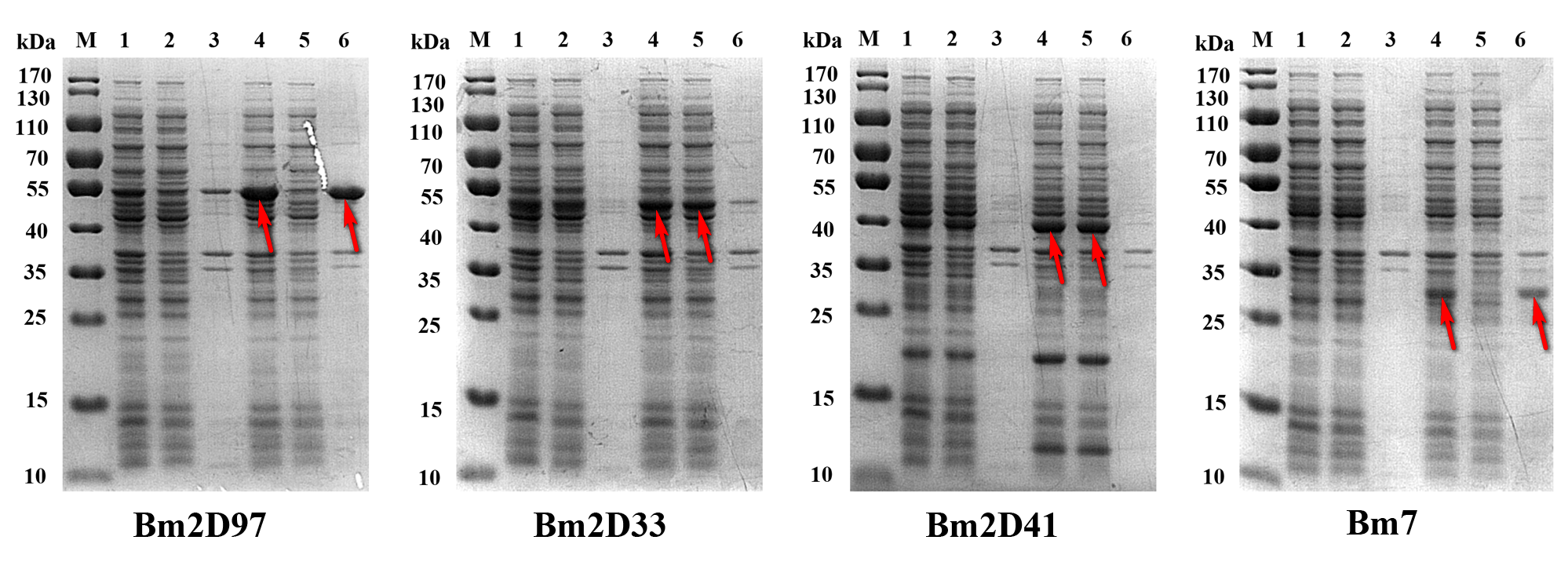

Supplement: Supplementary file 8 — Figure S2. Soluble expression analysis of rBm2D97, rBm2D33, rBm2D41 and rBm7. M: MW markers; Lane 1: pre-induction extraction of whole-cell protein; Lane 2: pre-induction supernatant; Lane 3: pre-induction precipitation; Lane 4: extraction of whole-cell proteinpost IPTG induction; Lane 5: supernatant post IPTG induction; Lane 6: precipitation post IPTG induction. Target proteins are marked with red arrow. (TIFF 1265 kb) [file 13071_2018_2951_MOESM8_ESM.tiff]

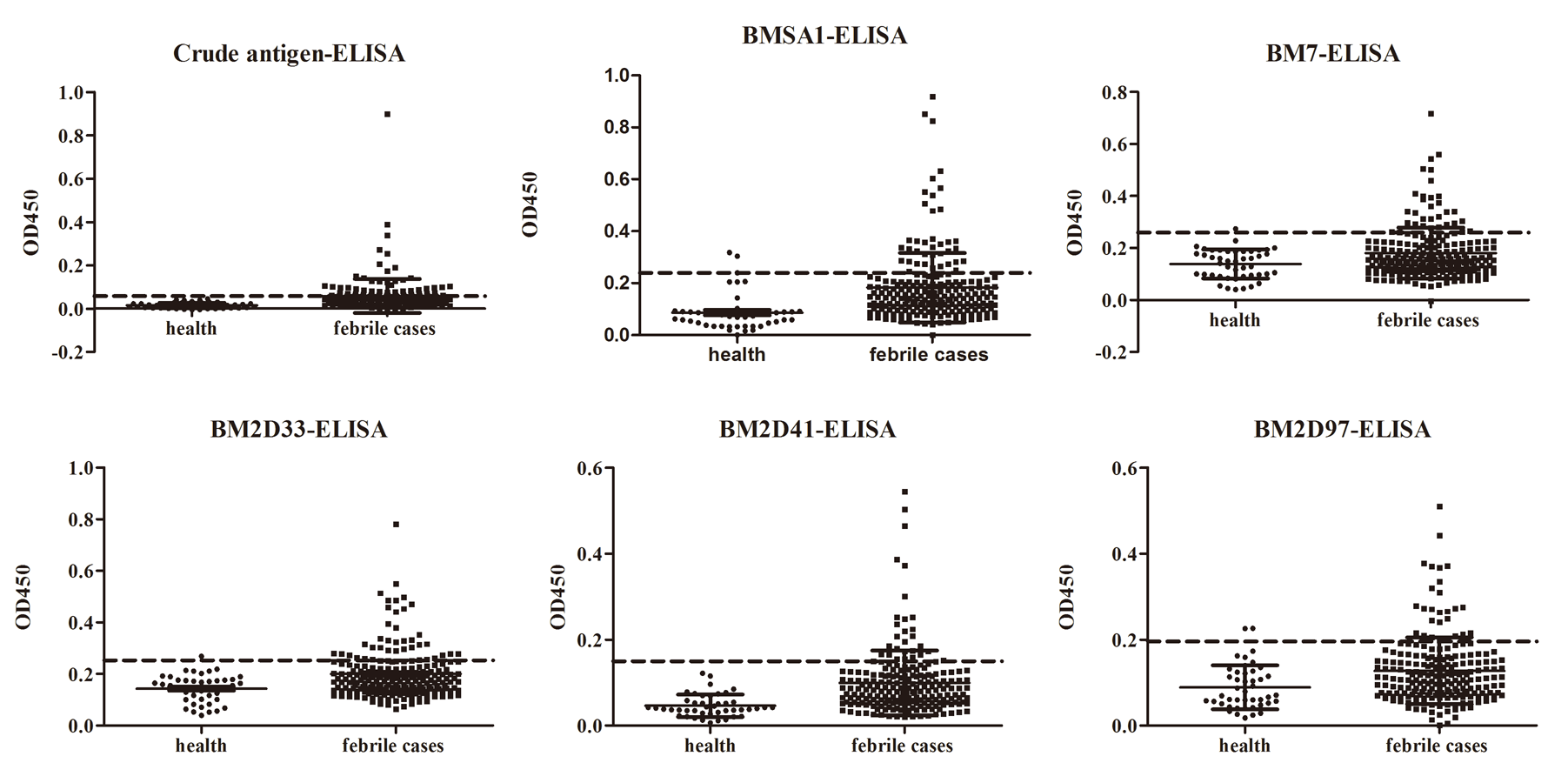

Supplement: Supplementary file 9 — Figure S3. Two hundred febrile cases evaluated by crude B.microti proteins and recombinant antigens. (TIFF 417 kb) [file 13071_2018_2951_MOESM9_ESM.tiff]

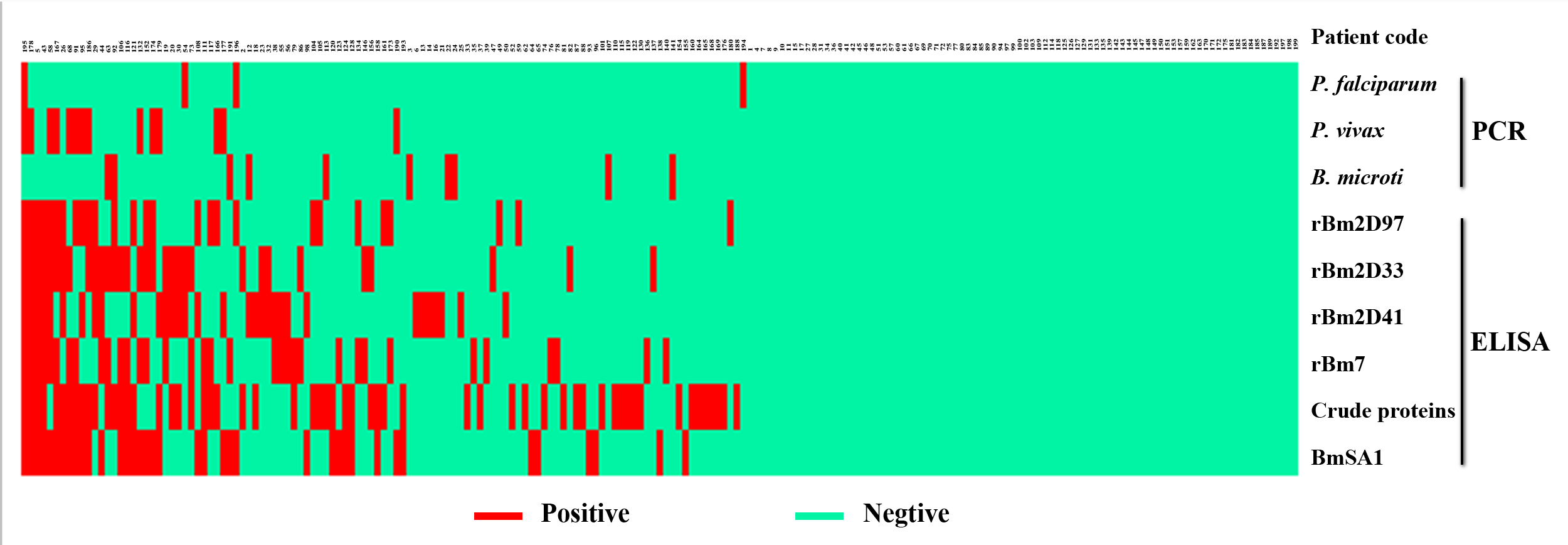

Supplement: Supplementary file 10 — Figure S4. Evaluating febrile cases using nested-PCR and recombinant proteins by ELISA. (TIF 6386 kb) [file 13071_2018_2951_MOESM10_ESM.tif]
