# Supplementary material for: Screening for biomarkers reflecting the progression of Babesia microti infection
Source: Parasit Vectors. 2018 Jul 3;11:379. doi: 10.1186/s13071-018-2951-0 (PMC6029176; doi:10.1186/s13071-018-2951-0)
Supplement: Supplementary file 7 — Table S6. Babesia microti proteins were grouped by immunoreactivity patterns. (DOCX 17 kb) [file 13071_2018_2951_MOESM7_ESM.docx]

**Additional file 6: Table S6. *B. microti* proteins were grouped by immunoreactivity patterns**

| **IgG** | | **IgM** | |
| --- | --- | --- | --- |
| Group 1 | 2D3 | Group 1 | 2D3 |
|  | 2D4 |  | 2D4 |
|  | 2D5 |  | 2D5 |
|  | 2D8 |  | 2D10 |
|  | 2D9 |  | 2D11 |
|  | 2D57 |  | 2D15 |
|  | 2D58 |  | 2D17 |
|  | 2D62 |  | 2D31 |
|  | 2D77 |  | 2D32 |
|  | 2D78 |  | 2D36 |
|  | 2D93 |  | 2D41 |
| Group 2 | 2D10 |  | 2D62 |
|  | 2D11 |  | 2D63 |
|  | 2D12 |  | 2D64 |
|  | 2D13 |  | 2D73 |
|  | 2D14 |  | 2D99 |
|  | 2D15 |  | 2D108 |
|  | 2D16 |  | 2D110 |
|  | 2D19 |  | 2D113 |
|  | 2D24 | Group 2 | 2D8 |
|  | 2D25 |  | 2D9 |
|  | 2D26 |  | 2D12 |
|  | 2D29 |  | 2D13 |
|  | 2D54 |  | 2D14 |
|  | 2D55 |  | 2D16 |
|  | 2D63 |  | 2D18 |
|  | 2D64 |  | 2D19 |
|  | 2D66 |  | 2D20 |
|  | 2D68 |  | 2D21 |
|  | 2D72 |  | 2D22 |
|  | 2D73 |  | 2D24 |
|  | 2D74 |  | 2D25 |
|  | 2D75 |  | 2D26 |
|  | 2D79 |  | 2D27 |
|  | 2D80 |  | 2D28 |
|  | 2D82 |  | 2D29 |
|  | 2D83 |  | 2D30 |
|  | 2D85 |  | 2D33 |
|  | 2D86 |  | 2D34 |
|  | 2D87 |  | 2D35 |
|  | 2D91 |  | 2D37 |
|  | 2D92 |  | 2D38 |
|  | 2D96 |  | 2D39 |
|  | 2D97 |  | 2D40 |
|  | 2D98 |  | 2D42 |
|  | 2D99 |  | 2D43 |
|  | 2D106 |  | 2D44 |
|  | 2D108 |  | 2D46 |
|  | 2D110 |  | 2D47 |
|  | 2D111 |  | 2D49 |
|  | 2D112 |  | 2D52 |
|  | 2D113 |  | 2D53 |
|  | 2D114 |  | 2D66 |
|  | 2D116 |  | 2D68 |
|  | 2D118 |  | 2D74 |
|  | 2D119 |  | 2D75 |
|  | 2D124 |  | 2D77 |
|  | 2D127 |  | 2D78 |
|  | 2D128 |  | 2D80 |
| Group 3 | 2D17 |  | 2D83 |
|  | 2D18 |  | 2D85 |
|  | 2D20 |  | 2D87 |
|  | 2D21 |  | 2D96 |
|  | 2D22 |  | 2D106 |
|  | 2D27 |  | 2D111 |
|  | 2D28 |  | 2D112 |
|  | 2D30 |  | 2D116 |
|  | 2D31 |  | 2D118 |
|  | 2D32 |  | 2D119 |
|  | 2D33 |  | 2D128 |
|  | 2D34 | Group 3 | 2D54 |
|  | 2D35 |  | 2D55 |
|  | 2D36 |  | 2D57 |
|  | 2D37 |  | 2D58 |
|  | 2D38 |  | 2D72 |
|  | 2D39 |  | 2D79 |
|  | 2D40 |  | 2D82 |
|  | 2D41 |  | 2D86 |
|  | 2D42 |  | 2D91 |
|  | 2D43 |  | 2D92 |
|  | 2D44 |  | 2D93 |
|  | 2D46 |  | 2D97 |
|  | 2D47 |  | 2D98 |
|  | 2D49 |  | 2D107 |
|  | 2D52 |  | 2D114 |
|  | 2D53 |  | 2D124 |
|  | 2D107 |  | 2D125 |
|  | 2D125 |  | 2D127 |
| Group 4 | B7 | Group 4 | B7 |
